# Prognostic Value of Programmed Cell Death 1 Ligand-1 in Patients With Bone and Soft Tissue Sarcomas: A Systemic and Comprehensive Meta-Analysis Based on 3,680 Patients

**DOI:** 10.3389/fonc.2020.00749

**Published:** 2020-06-02

**Authors:** Feng Wang, Tao Yu, Chengbin Ma, Hongmou Yuan, Haifei Zhang, Zhiyu Zhang

**Affiliations:** ^1^Department of Orthopedics, The Fourth Affiliated Hospital of China Medical University, Shenyang, China; ^2^Center for Translational Medicine, The Fourth Affiliated Hospital of China Medical University, Shenyang, China

**Keywords:** programmed cell death 1 ligand-1, sarcoma, meta-analysis, prognosis, overall survival

## Abstract

**Background:** Programmed cell death 1 ligand-1 (PD-L1) is an immune checkpoint molecule that acts to protect cancer cells from immune surveillance and is considered as a prognostic biomarker in several cancers, but the prognostic value of PD-L1 in bone and soft tissue sarcomas remains inconclusive. In the present meta-analysis, the clinicopathological and prognostic value of PD-L1 in sarcomas was evaluated.

**Method:** We performed a systemic and comprehensive meta-analysis by searching the PubMed, Medline, Cochrane Library, EMBASE, and Web of Science databases up to October 31, 2019. Eligible articles were incorporated, and pooled hazard ratios (HRs) and odds ratios (ORs) with their 95% confidence intervals (CIs) were used to estimate the outcomes.

**Results:** Thirty-six articles containing 39 independent studies with 3,680 bone and soft tissue sarcoma patients were included in our meta-analysis. The pooled results showed that PD-L1 overexpression could predict poor overall survival (HR 1.45, 95% CI 1.11–1.90, *P* < 0.01), metastasis-free survival (HR 1.58, 95% CI 1.14–2.19, *P* < 0.01), and event-free survival (HR 2.82, 95% CI 1.69–4.71, *P* < 0.01) in sarcomas. Furthermore, PD-L1 overexpression was correlated with a higher rate of tumor metastasis (OR 2.95, 95% CI 1.32–6.60, *P* < 0.01), a more advanced tumor grade (OR 3.63, 95% CI 2.55–5.16, *P* < 0.01), and more T lymphocyte infiltration (OR 5.55, 95% CI 2.86–10.76, *P* < 0.01). No obvious publication bias was observed, and the sensitivity analysis showed that our results were robust.

**Conclusion:** The results of our meta-analysis indicate that high PD-L1 expression might serve as a valuable and predictive biomarker for adverse clinicopathological features and poor prognosis in patients with sarcoma.

## Introduction

Bone and soft tissue sarcomas are a group of heterogeneous and rare neoplasms with more than 50 distinct histologic subtypes that originate from mesenchymal tissues, and these sarcomas account for <1% of adult cancers and 7–15% of pediatric malignancies (1). In recent years, due to the improvement of surgical techniques and the effective combining of chemotherapy or radiotherapy with surgery, a satisfactory but stagnant survival of over 60% has been achieved (2–4). Moreover, the response of sarcomas to conventional chemotherapy and radiotherapy varies, and for those patients with recurrent and metastatic disease, their survival remains unsatisfactory, with an average survival period of <1 year (5–7). Thus, novel treatment options for these patients are of utmost urgency.

Immunotherapy is considered as one of the foremost options for cancer treatment and has recently achieved tremendous success (8, 9). In the immune system, immune tolerance is an important process that inhibits the role of innate immunity in the cancer microenvironment. One of the immune tolerance mechanisms is the immune checkpoint mechanism. Immune checkpoint molecules, such as cytotoxic T lymphocyte antigen 4, lymphocyte activation gene 3, programmed cell death 1 (PD-1), and programmed cell death 1 ligand-1 (PD-L1), are expressed in the microenvironment of cancers (10), and suppressive signals are transmitted to T cells to prevent excessive immune responses. Thus, immune checkpoint inhibitors are emerging and becoming a prevailing form of systemic therapy regarded to “release the brakes” on the immune system. In malignant tumors, PD-L1 is often expressed on the surface of tumor cells and helps promote tumor evasion from the immune system by activating PD-1 and inhibiting the function of T cells. Immune checkpoint inhibitors targeting the PD-L1/PD-1 immune checkpoint axis have shown considerable success in cancers (11) but not in bone and soft tissue sarcomas (12–14). To investigate the underlying mechanism of PD-L1 in the progression of sarcomas and assess the clinical value of PD-L1, the correlation between PD-L1 expression and the clinical outcomes of sarcoma patients was assessed. However, more studies are emerging to show controversial results in the past several years (15–17). In addition, although several meta-analyses have been conducted to assess the prognostic value of PD-L1 in sarcomas, the results have been inconclusive (18, 19) due to the inconsistent results of PD-L1 assessment assays and the limited number of included studies.

In the present meta-analysis, the prognostic significance of PD-L1 expression was systemically and comprehensively evaluated with 39 independent studies of 3,680 bone and soft tissue sarcoma patients to investigate whether PD-L1 could serve as a prognostic biomarker in sarcomas.

## Materials and Methods

### Search Strategies

A systemic and comprehensive literature search was conducted in five databases with no language or publication date limitations: PubMed, Medline, Cochrane Library, EMBASE, and Web of Science. Articles published before October 2019 were included in the meta-analysis, and the last search was conducted on October 31, 2019. The following keywords were used to search the relevant literature: “sarcoma” or “bone sarcoma” or “soft tissue sarcoma” or “osteosarcoma” or “Ewing sarcoma” or “chondrosarcoma” or “myosarcoma” or “fibrosarcoma” or “synovial sarcoma” or “malignant fibrous histiocytoma” or “liposarcoma” or “epithelioid sarcoma” or “spindle cell sarcoma” or “carcinosarcoma” or “leiomyosarcoma” or “angiosarcoma” or “hemangiosarcoma” or “lymphangiosarcoma” or “malignant lymphangioendothelioma” and “PD-L1” or “B7-H1” or “CD274” or “programmed cell death 1 ligand 1 protein.” Moreover, we manually searched the references in each study to avoid missing potentially relevant data.

### Inclusion and Exclusion Criteria

The eligible studies included in the meta-analysis had to be in accordance with the following criteria: (1) studies on patients with pathologically confirmed bone and/or soft tissue sarcomas, (2) studies focusing on the correlations between PD-L1 expression and the survival and/or clinicopathological outcome(s), and (3) studies utilizing defined cutoff values to classify the PD-L1 expression as “high” and “low” or “positive” and “negative.” Studies were excluded if they (1) were reviews, case reports, letters, or conference abstracts, (2) investigated cell experiments and/or animal experiments, (3) were not related to PD-L1 expression, or (4) comprised overlapping patients and/or insufficient data. The eligibility of the included studies was determined by two authors independently. Any discrepancy was resolved by a consensus after a discussion.

### Data Extraction and Quality Assessment

The data of interest were extracted, including (1) basic characteristics (first author, publication year, tumor type, number and source of patients, presence of positive/high PD-L1 expression, cutoff value, assay method, biomarkers, and follow-up duration), (2) hazard ratios (HRs) with 95% confidence intervals (CIs) that either were given directly or could be calculated from published and available data by using the Cox regression method with a standard procedure (SPSS version 16.0) or Tierney's methods (20), and (3) data to estimate odds ratios (ORs) with 95% CIs for the correlations between PD-L1 and clinicopathological outcome(s). The HRs from the multivariate analysis were extracted if the HRs from both the univariate and the multivariate analysis were available.

The Newcastle–Ottawa Scale (NOS, https://www.ohri.ca/programs/clinical/epidemiology/oxford.asp) system was used to assess the methodological quality of each study, and the included studies were assessed for risk of bias by using the Quality and Prognosis Studies (QUIPS) method (21). The risk of bias was assessed for the following domains: study participation, study attrition, prognostic factor measurement, outcome measurement, study confounding, and statistical analysis. The score was classified and reported as low, moderate, or high. The assessment was performed by two authors independently. If there was any disagreement, it was resolved by a consensus after a discussion.

### Statistical Analysis

Estimates of HRs, ORs, and their 95% CIs were used to assess the pooled survival related to and the clinicopathological significance of PD-L1 expression. Cochran's Q test and Higgins *I*^2^ statistic were employed to assess the statistical heterogeneity among studies. A fixed-effects model was appropriately used if there was no significant heterogeneity (*P* > 0.10 or *I*^2^ <50%); otherwise, a random-effects model was used, and a subgroup meta-analysis was conducted to identify the underlying heterogeneity when at least five studies were included. Publication bias was assessed by using a funnel plot analysis, Begg's tests, and Egger's tests, and a sensitivity analysis was used to assess the stability of the pooled results when at least five studies were included. *P* < 0.05 was considered as statistically significant. All analyses were conducted by using Stata version 14.0 (Stata Corporation, College Station, TX, USA).

## Results

### Study Selection

**Figure 1:**
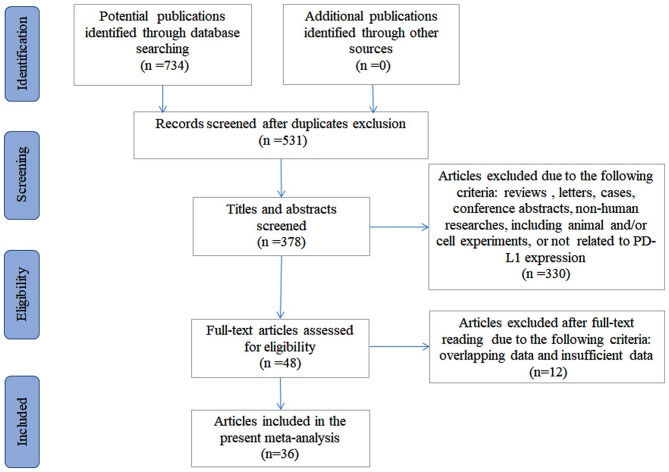
Flow diagram of the study selection process.

There were 734 articles identified initially from the systemic and comprehensive search. After the duplicates were excluded, 531 articles were subjected to further screening. After viewing the title and the abstract, 378 articles remained. Three hundred thirty articles were omitted due to irrelevance. After a full-text review, 12 articles were excluded, including one with overlapping data and 11 with insufficient data. In total, 36 articles containing 39 independent studies with 3,680 bone and soft tissue sarcoma patients were included (15–17, 22–54), among which 31 articles were used to evaluate the prognostic significance of PD-L1 expression and 27 articles were used to evaluate the clinicopathological significance. The selection process is shown in Figure 1.

### Study Characteristics and Quality Assessment

The summarized study characteristics are shown in Table 1. Briefly, the publication year of the eligible studies, written in English, ranged from 2013 to 2019. The patients were collected internationally from Asian (*n* = 17) to non-Asian (*n* = 23) populations. The number of patients ranged from 11 to 492. A variety of sarcomas were investigated in the studies, including osteosarcoma (*n* = 11), soft tissue sarcoma (STS, *n* = 9), unclassified sarcoma (*n* = 4), angiosarcoma (*n* = 4), leiomyosarcoma (*n* = 3), liposarcoma (*n* = 2), chondrosarcoma (*n* = 2), Ewing's sarcoma (*n* = 1), myeloid sarcoma (*n* = 1), pleomorphic sarcoma (*n* = 1), and synovial sarcoma (*n* = 1). Immunohistochemistry (IHC), quantitative polymerase chain reaction (qPCR), or RNA sequencing methodologies were employed to evaluate the PD-L1 expression. The cutoff values were available in studies using IHC, and the positivity ranged from 5.90 to 75.90%. Moreover, data from studies were extracted to analyze the HRs of overall survival (OS, *n* = 30), as a primary endpoint, and other survival outcomes, including recurrence-free survival (RFS, *n* = 4), metastasis-free survival (MFS, *n* = 3), event-free survival (EFS, *n* = 3), disease-free survival (DFS, *n* = 5), and progression-free survival (PFS, *n* = 4). The follow-up duration ranged from 1 to 366 months based on the available data. The correlation between PD-L1 expression and clinicopathological outcomes was assessed with data from 29 studies, including patient age and sex, tumor size, depth and localization, tumor grade and stage, metastasis, recurrence, previous chemotherapy/radiotherapy, chemotherapy response, tumor-infiltrating lymphocytes (TILs), and programmed cell death 1 (PD1) positivity.

**Table 1:** Characteristics of the included studies.

**Study**	**References**	**Patients**	**Tumor type**	**Positive (%)**	**Source**	**Cutoff value**	**Assay method**	**Outcome**	Biomarker^*^	**HR source (M/U)**	**Follow-up (m)**
1	(17)	29	Angiosarcoma	22 (75.90)	Japan	>5% of cells	IHC	OS, CPF	PD-L1, PD1	SC (U)	NA
2	(23)	56	Liposarcoma	13 (23.20)	China	IRS >0	IHC	CPF	PD-L1, PD1, CD4, CD8, FoxP3, and CD20	NA	NA
3	(22)	19	Osteosarcoma	8 (42.10)	Japan	NA	qPCR	OS, MFS	PD-L1, GZMB, PRF, and IFNγ	SC (U)	8.2–237.4
4	(15)	25	Angiosarcoma	5 (19.00)	USA	≥5% of cells	IHC	OS	PD-L1, VEGF, and VEGFR	Directly (M)	NA
5	(16)	128	STS	23 (40.40)	Germany	≥0% of TCs	IHC	OS, DFS	PD-L1, PD-1, CD3, and CD8	Directly (M)	2–222
6	(33)	11	Leiomyosarcoma	4 (36.40)	Israel	>1% of TCs	IHC	OS,CPF	MMR proteins, PD-L1, PD-1, CD3, and CD8	Cox-regression (M)	4–120
7	(32)	131	MS	10 (10.20)	Japan	≥1% of TCs	IHC	OS, PFS	PD-L1, PD1, TP53, and CXCR4	SC (U)	8 (1–100)
8	(31)	106	Leiomyosarcoma	32 (30.20)	Netherlands	≥1% of cells	IHC	OS, DFS, CPF	CD163, CD3, PD-L1/PD-L2, and HLA-I	SC (U)	NA
9	(30)	370	Ewing's sarcoma	71 (19.20)	Spain	>5% of TCs	IHC	OS,PFS, CPF	PD-L1, PD-1, and CD8	*P* value (U)	NA
10	(29)	36	SS	9 (47.40)	Japan	NA	qPCR	OS, PFS	CD4, CD8, FOXP3, CD163, HLA, PD-L1, and PD-L2	SC(U)	113 (6–366)
11	Cohort a (28)	32	Liposarcoma	7 (21.90)	Korea	≥1% of TCs	IHC	OS, RFS, CPF	PD-L1	SC(U)	19
12	Cohort b (28)	60	PS	12 (20.00)	Korea	≥1% of TCs	IHC	OS, RFS, CPF	PD-L1	SC (U)	49
13	(27)	46	STS	21 (45.70)	USA	>1% of cells	IHC	MFS,CPF	PD-L1	SC (U)	69.8
14	(26)	59	Chondrosarcoma	40 (67.80)	China	≥1% of cells	IHC	RFS, CPF	PD-L1, PD-L2, Ki67, and TP53	Directly (M)	20 (1–118)
15	(25)	92	Osteosarcoma	65 (70.70)	China	≥1% of cells	IHC	CPF	PD-L1	NA	NA
16	(24)	234	Sarcomas	45 (20.50)	China/USA	≥10% of cells	IHC	OS, CPF	PD-L1, PD-L2, and PD-1	SC (U)	NA
17	(44)	492	STS	196 (39.80)	France	NA	RNA seq	MFS,CPF	PD-L1	Directly (M)	0–120
18	(43)	24	Angiosarcoma	16 (66.70)	Italy	≥5% of TCs	IHC	OS, DFS, CPF	PD-L1	SC (U)	NA
19	Cohort a (42)	256	STS	34 (21.10)	Germany	NA	RNA seq	OS	CD3D, CD4/CD8, PD-L1, and CD3Z	Directly (U)	NA
20	Cohort b (42)	103	Leiomyosarcoma	16 (16.00)	Germany	NA	RNA seq	OS	CD3D, CD4, CD68, CD4/CD8, PD-L1, and CD3Z	Directly (U)	NA
21	(41)	13	Osteosarcoma	9 (69.20)	Brazil	IRS > 2	IHC	OS, CPF	HLA-G, HLA-E, and PD-L1	Cox-regression (M)	32 (2–156)
22	(40)	72	Osteosarcoma	30 (41.70)	USA	Score ≥ 2	IHC	OS, CPF	PD-L1	Directly (M)	NA
23	(39)	86	Osteosarcoma	12 (14.00)	Italy	>5% of cells	IHC	OS	CD8, Tia1,CD3, FOXP3, PD-1, PD-L1, Argase-1, CD303, CD68, and CD163	Directly (M)	96 (12–156)
24	(38)	81	STS	48 (59.00)	USA	IHC score >0	IHC	CPF	PD-L1 and PD-1	NA	NA
25	(37)	163	STS	19 (11.70)	China	>1% of TCs	IHC	OS, DFS, CPF	PD-L1 and FOXP3	Directly (U)	75 (1–176)
26	(36)	22	Osteosarcoma	4 (18.20)	Netherlands	≥1% of cells	IHC	OS, DFS, CPF	HLA-I, CD3, CD8, and PD-L1	SC (U)	56 (14–117)
27	(35)	208	Sarcomas	18 (8.65)	Netherlands	≥10% of TCs	IHC	OS	PD-1, PD-L1, and CD8	SC (U)	NA
28	(34)	93	Osteosarcoma	33 (35.50)	China	≥10% of cells	IHC	OS, CPF	VEGFR2 and PD-L1	SC (U)	NA
29	(49)	106	Angiosarcoma	32 (30.20)	Japan	>5% of cells	IHC	OS, CPF	PD-L1 and PD1	Directly (M)	20 (3–100)
30	(48)	82	STS	35 (42.70)	Korea	Score ≥ 2	IHC	OS,RFS,CPF	PD-L1	Directly (M)/SC (U)	34 (4–85)
31	Cohort a (47)	51	Osteosarcoma	3 (5.90)	USA	≥1% of cells	IHC	EFS, CPF	PD-L1	SC (U)	84 (4–150)
32	Cohort b (47)	41	Osteosarcoma	12 (29.30)	USA	≥1% of cells	IHC	EFS, CPF	PD-L1, CD68, and CD1a	SC (U)	54 (15–100)
33	(46)	22	Chondrosarcoma	9 (41.00)	Netherlands	≥1% of cells	IHC	OS	PD-L1	SC (U)	54 (15–100)
34	(45)	66	Sarcomas	20 (30.30)	Turkey	>5% of cells	IHC	OS,PFS, CPF	PD-1 and PD-L1	Cox-regression (M)	30 (4–310)
35	(52)	59	Sarcomas	36 (59.30)	UK	>5% of TCs	IHC	CPF	PD-L1 and CD8/PD1	NA	33 (3–200)
36	(51)	47	STS	4 (8.50)	USA	>1% of TCs	IHC	OS,CPF	CD3, CD4, CD8, FOXP3, PD-L1, and PD1	P value (U)	NA
37	(50)	16	Osteosarcoma	12 (75.00)	USA	>1 cell /HPF	IHC	CPF	PD-L1 and PD1	NA	NA
38	(53)	38	Osteosarcoma	9 (27.00)	USA	Score >2-log	qPCR	OS,CPF	PD-L1	SC (U)	36 (1–200)
39	(54)	105	STS	68 (64.80)	Korea	Score ≥8	IHC	OS, EFS, CPF	PD1 and PD-L1	Directly (M)	35 (1–175)

* Analyzed for survival and/or clinicopathological outcomes.

*HR, hazard ratio; IHC, immunohistochemistry; qPCR, quantitative polymerase chain reaction; OS, overall survival; EFS, event-free survival; MFS, metastasis-free survival; PFS, progression-free survival; RFS, recurrence-free survival; DFS, disease-free survival; CPF, clinicopathological feature; STS, soft tissue sarcomas; MS, myeloid sarcoma; SS, synovial sarcoma; PS, pleomorphic sarcoma; SC, survival curve; IRS, immunoreactive score; NA, not available; TC, tumor cell; HPF, high-power field; U, univariate; M, multivariate; PD-L1, programmed cell death 1 ligand-1; PD-L2, programmed cell death 1 ligand-2; PD1, programmed cell death 1; VEGF, vascular endothelial growth factor; VEGFR, vascular endothelial growth factor receptor; MMR, mismatch repair; HLA, human leukocyte antigen; CXCR4, CXC chemokine receptor 4; TP53, tumor suppressor p53; GZMB, granzyme B; PRF, perforin; IFNγ, interferon-γ; FOXP3, forkhead box P3; Tia1, cytotoxic granule-associated RNA binding protein 1*.

The included 39 studies were critically appraised for methodological quality and risk of bias by using the NOS system and the QUIPS criteria, respectively. The results showed that all of the studies were of high methodological quality, with scores that ranged from six to nine stars. Most of the studies were retrospective studies and were assessed as having a low or moderate risk of bias. Almost no study provided information about patients lost to follow-up. Due to a lack of sufficient data on survival outcome(s) or study confounding, studies with only clinicopathological features were scored with a high risk of bias. Some studies were scored with a high or moderate risk of study participation due to missing details and the limited number of patients. A few studies were scored with a high or moderate risk of study confounding because of insufficient survival analyses for some important factors. The results are shown in Supplementary Table 1.

### Prognostic Value of PD-L1 Expression in Sarcomas

**Figure 2:**
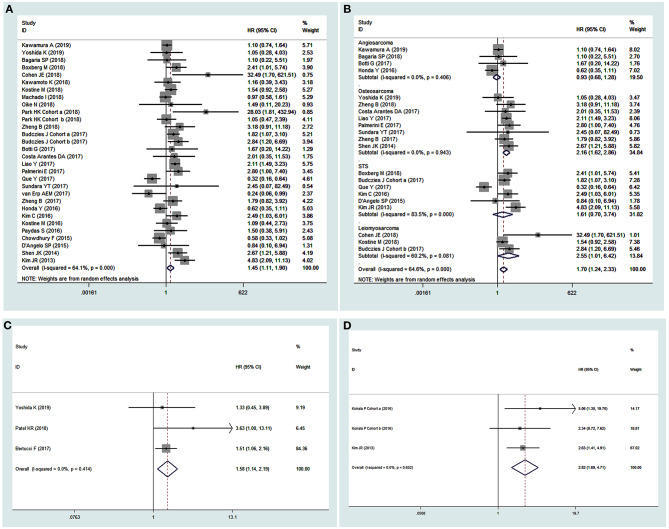
Forest plots of pooled HR for pooled overall survival (OS) **(A)** and OS stratified by tumor subtypes **(B)**, metastasis-free survival **(C)**, and event-free survival **(D)**.

A random-effects model was used in the analysis for OS as obvious heterogeneity was observed (*I*^2^ = 64.10%, *P* = 0.00). The results showed that elevated PD-L1 expression was correlated with poor OS (HR 1.45, 95% CI 1.11–1.90, *P* < 0.01, Figure 2A). Moreover, to investigate the underlying source of heterogeneity, subgroup analyses by tumor type, publication date, sample size and source, analysis model of effects, PD-L1 positivity, assay method, and PD-L1 expression region (in the tumor or the tumor microenvironment) were conducted. The results showed that none of these factors were the underlying source of heterogeneity, and the overall value of PD-L1 did not change (Table 2). Moreover, as various tumor subtypes were observed in the present analysis, a stratified analysis by tumor subtype was conducted, in which no less than three studies were included. A random-effects model was used (*I*^2^ = 64.60%, *P* = 0.00), and the results showed that PD-L1 overexpression could predict poor OS specifically in osteosarcoma (*P* < 0.01) and leiomyosarcoma (*P* < 0.05, Figure 2B). Thus, it was indicated that PD-L1 was a predictor for poor OS in sarcoma patients.

**Table 2:** Summary of correlation between PD-L1 expression and survival outcomes.

**Subgroup**	**Study (*n*)**	**HR (95% CI)**	***P***	**Heterogeneity**		**Effects model**
				***I*^**2**^ (%)**	***P***	
OS	30	1.45 (1.11–1.90)	<0.01^*^	64.10	0.00	Random effects
**Tumor type**						
Bone sarcoma	10	1.72 (1.30–2.28)	<0.01^*^	14.30	0.31	
Non-bone sarcoma	20	1.33 (0.92–1.93)	0.14	70.40	0.00	
**Publication (year)**						
≥2018	12	1.41 (1.02–1.94)	<0.05^*^	31.70	0.14	
<2018	18	1.40 (0.94–2.09)	0.10	73.60	0.00	
**Sample source**						
Asian	14	1.51 (0.93–2.44)	0.10	71.90	0.00	
Non-Asian	16	1.48 (1.08–2.01)	<0.05^*^	52.60	0.01	
**Sample size (*****n*****)**						
≥85	12	1.32 (0.87–2.01)	0.19	78.50	0.00	
<85	18	1.55 (1.09–2.21)	0.01^*^	42.40	0.03	
**Analysis model**						
Univariate	21	1.27 (0.93–1.72)	0.13	59.50	0.00	
Multivariate	9	2.01 (1.17–3.45)	<0.05^*^	66.90	0.00	
**Positivity (%)**						
≥30	15	1.62 (1.13–2.33)	<0.01^*^	65.90	0.00	
<30	15	1.28 (0.83–1.96)	0.26	63.70	0.00	
**Assay method**						
IHC	26	1.36 (1.00–1.86)	<0.05^*^	67.10	0.00	
Non-IHC	5	2.06 (1.42–2.98)	<0.01^*^	0.00	0.00	
**Expression region**						
Tumor cell	14	1.32 (0.772–2.27)	0.31	74.70	0.00	
Non-tumor cell	16	1.59 (1.24–2.03)	<0.01^*^	34.80	0.08	
RFS	4	1.42 (0.92–2.21)	0.12	0.00	0.45	Fixed effects
MFS	3	1.58 (1.14–2.19)	<0.01^*^	0.00	0.41	Fixed effects
EFS	3	2.82 (1.69–4.71)	<0.01^*^	0.00	0.65	Fixed effects
DFS	5	1.17 (0.53–2.58)	0.70	74.5	0.00	Random effects
**Publication date**						
≥2018	2	1.39 (0.66–2.91)	0.39	47.30	0.17	
<2018	3	1.09 (0.27–4.46)	0.90	75.80	0.12	
**Sample size**						
≥100	3	0.90 (0.35–2.34)	0.83	83.20	0.00	
<100	2	2.29 (0.77–6.76)	0.14	0.00	0.84	
**Tumor type**						
STS	2	0.89 (0.16–4.80)	0.89	90.40	0.00	
Non-STS	3	1.24 (0.72–2.14)	0.44	0.00	0.43	
**Positivity (%)**						
≥35	2	2.27 (1.01–5.09)	0.05	0.00	0.84	
<35	3	0.82 (0.34–2.01)	0.66	76.00	0.02	
**Cutoff value**						
≥1% of tumor cells	3	2.27 (1.01–5.09)	0.66	0.00	0.84	
Non-≥1% of tumor cells	2	0.82 (0.34–2.01)	<0.05^*^	76.00	0.02	
**Expression region**						
Tumor cell	3	1.16 (0.28–4.75)	0.84	84.10	0.00	
Non-tumor cell	2	1.16 (0.66–2.04)	0.62	0.00	0.33	
PFS	4	1.00 (0.65–1.53)	0.99	19.8	0.29	Fixed effects

* Significant difference.

*HR, hazards ratio; CI, confidence interval; OS, overall survival; RFS, recurrence-free survival; MFS, metastasis-free survival; EFS, event-free survival; DFS, disease-free survival; PFS, progression-free survival; STS, soft tissue sarcoma; IHC, immunohistochemistry*.

Other extracted important survival outcomes in the present analysis, including RFS, MFS, PFS, EFS, and DFS, are survival parameters related to disease according to their definition, but these data had limited numbers and thus insufficient effect size. In our analysis, the results showed that an elevated PD-L1 expression predicted poor MFS (HR 1.58, 95% CI 1.14–2.19, *P* < 0.01, Figure 2C) and EFS (HR 2.82, 95% CI 1.69–4.71, *P* < 0.01, Figure 2D) but was not associated with other survival outcomes (Table 2). As obvious heterogeneity was observed (*I*^2^ = 74.50%, *P* = 0.003) in the DFS analysis, a random-effects model was used, and subgroup analyses were conducted. The results of the subgroup analyses showed that the heterogeneity was not reduced by stratification of the data by publication date, tumor type, sample size, PD-L1 positivity, cutoff value, and PD-L1 expression region. However, the pooled HR did not change, indicating that the result was stable. In addition, although stratified analyses by tumor type (bone sarcoma vs. non-bone sarcoma) were conducted for other survival outcomes, no conclusive and reliable results were observed due to the limited number of included studies, as shown in Supplementary Table 2.

### Clinicopathological Value of PD-L1 Expression in Sarcomas

The clinicopathological value of PD-L1 expression is summarized in Table 3. Briefly, 29 studies were included, and comprehensive meta-analyses were conducted to determine the correlation between the PD-L1 expression and patient age (*n* = 4) and sex (*n* = 21), tumor size (*n* = 6), depth (*n* = 3) and localization (*n* = 8), tumor grade (*n* = 15) and stage (*n* = 8), metastasis (*n* = 11), recurrence (*n* = 7), previous chemotherapy/radiotherapy (*n* = 10 and *n* = 7, respectively), chemotherapy response (*n* = 5), and TILs (*n* = 12). A random-effects model or a fixed-effects model was used based on the results of the heterogeneity analysis for each parameter, as shown in Table 3. PD-L1 overexpression was positively correlated with a higher rate of metastasis (OR 2.95, 95% CI 1.32–6.60, *P* < 0.01), a higher tumor grade (OR 3.63, 95% CI 2.55–5.16, *P* < 0.01), and more TILs (OR 5.55, 95% CI 2.86–10.76, *P* < 0.01) but was not correlated with other clinicopathological outcomes. For the TIL subset analysis, the PD-L1 expression was not correlated with CD4-positive TILs (OR 2.45, 95% CI 0.69–8.73, *P* = 0.17). Moreover, a stratified analysis by bone sarcoma vs. non-bone sarcoma was conducted for each outcome to clarify whether the correlation was tumor type dependent. The results showed that PD-L1 was specifically correlated with metastasis and CD8+ TILs in bone sarcomas and CD3+ TILs in non-bone sarcomas. In addition, PD-L1 overexpression was correlated with a good response to chemotherapy in bone sarcomas but with a poor response in non-bone sarcomas (Supplementary Table 3). For the outcomes with obvious heterogeneity, subgroup analyses were conducted. The results showed that some factors might be the underlying source of heterogeneity in some of the clinicopathological outcomes, as shown in Supplementary Table 4.

**Table 3:** Summary of correlation between PD-L1 expression and clinicopathological outcomes.

**Category**	**Study (*n*)**	**Heterogeneity**		**Effects model**	**OR (95% CI)**	***P*-value**
		***I*^**2**^ (%)**	***P***			
<60 years/≥60 years	4	0.00	0.64	Fixed	0.65 (0.39–1.10)	0.11
Male/female	21	19.00	0.21	Fixed	1.07 (0.88–1.30)	0.50
Size 5 cm/ ≤ 5 cm >10 cm/ ≤ 10 cm	6 3 3	54.40 57.20 54.40	0.05 0.10 0.05	Random Random Random	1.14 (0.54–2.42) 0.86 (0.29–2.50) 1.54 (0.48–4.97)	0.74 0.78 0.47
Depth	3	65.70	0.05	Random	1.60 (0.63–4.06)	0.33
Extremity/non-extremity	8	52.30	0.04	Random	1.01 (0.58–1.75)	0.99
Stage (high/low)	8	58.50	0.02	Random	1.94 (0.70–5.39)	0.20
Grade (high/low)	15	17.2	0.26	Fixed	3.63 (2.55–5.16)	<0.01^*^
Radiotherapy (Y/N)	7	66.60	0.01	Random	1.36 (0.58–3.20)	0.48
Chemotherapy (Y/N)	10	0.00	0.95	Fixed	1.06 (0.70–1.59)	0.79
Met/non-met	11	75.80	0.00	Random	2.95 (1.32–6.60)	<0.01^*^
Rec/non-rec	7	51.30	0.06	Random	2.08 (0.87–4.96)	0.10
CR (good/poor)	5	86.70	0.00	Random	0.55 (0.09–3.53)	0.53
TIL (Y/N)	12	68.90	0.00	Random	5.55 (2.86–10.76)	<0.01^*^
PD1 + TIL (Y/N)	5	0.00	0.41	Fixed	3.21 (1.85–5.58)	<0.01^*^
CD3 + TIL (Y/N)	5	0.00	0.51	Fixed	3.12 (1.49–6.54)	<0.01^*^
CD4 + TIL (Y/N)	2	0.00	0.94	Fixed	2.45 (0.69–8.73)	0.17
CD8 + TIL (Y/N)	7	70.10	0.00	Random	6.90 (2.62–18.20)	<0.01^*^
FOXP3 + TIL (Y/N)	2	0.00	0.62	Fixed	14.28 (4.47–45.65)	<0.01^*^

* Statistically significant.

*OR, odds ratio; CI, confidence interval; Met, metastasis; Rec, recurrence; CR, chemotherapy response; TIL, tumor-infiltrating lymphocyte; PD1, programmed cell death 1*.

### Publication Bias and Sensitivity Analysis

**Figure 3:**
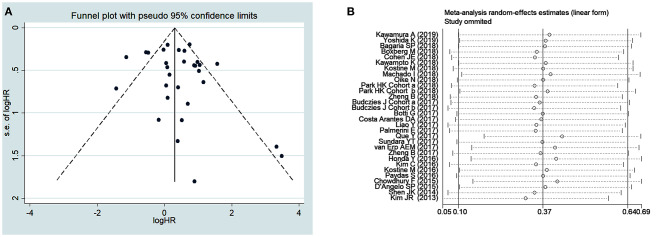
Funnel plots for publication bias **(A)** and sensitivity analysis **(B)** of overall survival.

Publication bias was assessed by using funnel plot analysis (Figure 3) and Begg's and Egger's tests (Table 4). For survival outcomes, no obvious publication bias was observed in the evaluation of OS (Figure 3A) or RFS, MFS, EFS, and DFS (Table 4), but publication bias was noted for PFS (*P* = 0.04 in Egger's tests). For clinicopathological outcomes, Egger's tests showed that there might be a publication bias in the analysis of PD1+ TILs (*P* = 0.03). No obvious publication bias was observed in any other analysis of clinicopathological outcomes.

**Table 4:** Begg's test and Egger's test for publication bias.

**Analysis value**	**Study (*n*)**	**Begg's test**		**Egger's test**	
		***z***	***P***	***t***	***P***
OS	30	0.82	0.41	1.10	0.28
RFS	4	1.02	0.31	1.18	0.36
MFS	3	1.04	0.30	0.73	0.60
EFS	3	1.04	0.30	0.69	0.62
DFS	5	0.73	0.46	1.98	0.14
PFS	4	1.70	0.09	5.06	0.04^*^
Age	4	0.34	0.73	2.78	0.11
Gender	21	0.27	0.79	−0.41	0.69
Size	6	0.00	1.00	0.00	1.00
Radiotherapy	7	0.00	1.00	0.56	0.60
Chemotherapy	10	1.07	0.28	−0.76	0.47
Localization	8	0.37	0.71	−0.32	0.76
Depth	3	1.04	0.30	−3.64	0.17
Grade	15	0.89	0.37	1.39	0.19
Stage	8	0.62	0.54	−0.08	0.94
Metastasis	11	0.00	1.00	0.41	0.69
Recurrence	7	1.50	0.13	0.70	0.52
CR	5	0.24	0.81	−0.09	0.93
TIL (Y:N)	12	0.07	0.95	−0.27	0.79
PD1 + TIL (Y:N)	5	1.22	0.22	4.10	0.03^*^
CD3 + TIL (Y:N)	5	−0.24	1.00	1.44	0.25
CD4 + TIL (Y:N)	2	0.00	1.00	–	–
CD8 + TIL (Y:N)	7	0.00	1.00	−0.31	0.77
FOXP3 + TIL (Y:N)	2	0.00	1.00	–	–

* Significant difference.

*OS, overall survival; RFS, recurrence-free survival; MFS, metastasis-free survival; EFS, event-free survival; DFS, disease-free survival; PFS, progression-free survival; CR, chemotherapy response; TIL, tumor-infiltrating lymphocyte; PD1, programmed cell death 1*.

A sensitivity analysis was performed to assess the effects of each individual study on the pooled HRs and ORs of our analysis. The results of the sensitivity analysis, which was performed by omitting one study at a time, showed that no unique study significantly affected the pooled HRs for OS (Figure 3B) and other outcomes (Supplementary Figures 1–14), indicating that the results of the present meta-analysis were stable and credible.

## Discussion

PD-L1, one of the immune checkpoint molecules, can inhibit T cell activation and proliferation by binding with PD-1 and protect tumor cells from the host immunologic surveillance system. Immunotherapy, by administration of immune checkpoint inhibitors, has been considered as the foremost method in individualized medicine due to its tremendous success in cancer treatment. Clinical trials have shown satisfactory results by using anti-PD-1/PD-L1 blockade in some cancers (11). However, the results in bone and soft tissue sarcomas are unsatisfactory (14). Recent studies have indicated that the high expression of PD-L1 is associated with poor prognosis in some cancers (55, 56). Moreover, several meta-analyses have evaluated the clinical significance of PD-L1 expression in patients with sarcomas, but the results have been inconclusive (18, 19). In the present meta-analysis, 39 independent studies (including 3,680 patients) were included, and the pooled results showed that PD-L1 overexpression could predict poor OS, MFS, and EFS. Furthermore, an elevated PD-L1 expression was correlated with a higher tumor metastasis rate, a more advanced tumor grade, and more T lymphocyte infiltration.

In terms of survival outcomes, the results showed that PD-L1 overexpression could predict poor OS and EFS, which was similar to the findings of previous reports (18, 19). As obvious heterogeneity was observed for OS, a random-effects model was used to reduce heterogeneity, and subgroup analyses were conducted. Although the results did not identify the underlying source of heterogeneity, the pooled results did not change in each subgroup analysis. Moreover, a stratified analysis by tumor subtype showed that PD-L1 overexpression could specifically predict poor OS in osteosarcoma and leiomyosarcoma. There was no obvious publication bias, and the sensitivity analysis indicated that the results were stable. Therefore, our results were robust and reliable. Some other factors should be considered to explain the underlying heterogeneity. There are more than 50 different subtypes of sarcoma, and more than 10 subtypes were included in the present analysis, which introduced heterogeneity into the whole meta-analysis. Moreover, differences in the detection techniques, such as antibody selection and dilution, and the antigen retrieval methods may have affected the sensitivity of detection, and the positivity of the PD-L1 expression might have been affected by the different cutoff values. In addition, the region used to define the PD-L1 expression was different among the included studies. These might be other explanations for the heterogeneity.

It was initially reported that PD-L1 overexpression predicted poor MFS but was not associated with DFS, RFS, or PFS in patients with sarcoma. A previous meta-analysis showed a similar result that the PD1/PD-L1 expression was associated with lymph node metastasis in patients with osteosarcoma (57). PD-L1 is widely considered to function in the tumor immunologic surveillance system; however, the tumor-intrinsic roles of PD-L1 in regulating the ability of human cancers to disseminate and to metastasize are currently not understood. PD-L1 is suggested to be involved in cancer metastasis via several mechanisms. PD-L1 is shown to promote epithelial-to-mesenchymal transition and is involved in tumor proliferation, migration, and invasion (58, 59). Moreover, cancer stem cells (CSCs) are considered to be involved in tumor metastasis (60), and PD-L1 is suggested to sustain stemness by promoting OCT4 and Nanog expression in CSCs (61) and promoting tumor-initiating cell generation and virulence in cancer (62). Thus, it is suggested that PD-L1 is involved in the possible immune evasion mechanism employed by CSCs during metastasis (63). In addition, several signaling pathways have been suggested to modulate the role of PD-L1 in cancer initiation, metastasis, and chemoresistance, including the MAPK/ERK pathway, the PI3K/AKT pathway, and the RAS/MEK pathway (64, 65). Sarcomas are a group of malignancies with a high propensity for metastasis, and the prognosis of metastatic patients remains poor. Further studies are encouraged to investigate the mechanism by which PD-L1 is involved in sarcoma metastasis. However, as there were only three studies in the pooled analysis for MFS, the results should be interpreted with caution.

The results from the analyses of clinicopathological outcomes were similar to those from previous reports (18, 19). An elevated PD-L1 expression was correlated with a higher rate of metastasis, a higher tumor grade, and more T lymphocyte infiltration. In the analysis for tumor grade, the result was different from a previous report (19). In other cancers, the correlation between PD-L1 expression and tumor grade remains controversial (55, 66), which might arise from the inherent differences between cancers. Moreover, the prognosis of patients with high-grade sarcomas is not satisfactory. It was suggested that PD-L1 overexpression might predict the presence of advanced sarcoma. In addition, our results might be more conclusive and reliable than those from other meta-analyses because more studies were included (*n* = 15). Moreover, although the results showed that PD-L1 expression was not correlated with chemotherapy response, stratified analyses by tumor type showed that PD-L1 overexpression might predict good response to chemotherapy in bone sarcomas but poor response in non-bone sarcomas, and this difference might be due to the difference in chemotherapy sensitivity between these two types of sarcoma.

Another major result in our analysis was that high PD-L1 expression was correlated with more TILs, which was similar to the findings of previous analyses in sarcomas and some other cancers (19, 67, 68). However, in the analyses of T cell subtype, the results showed that elevated PD-L1 expression was mainly correlated with PD1+ T cells, CD3+ T cells, and CD8+ T cells but not with CD4+ T cells. In the process of tumor immune tolerance, the PD1/PD-L1 axis acts to suppress the T cell response. PD-L1 is expressed on tumor cells and binds with PD-1, which is expressed on tumor-infiltrating CD8+ T cells as well as CD4+ T cells and other inflammatory cells. Additionally, PD-L1 makes tumor cells less susceptible to specific CD8+ T cells (69). Therefore, the killing effect of T cells on tumor cells is inhibited (70, 71). Moreover, PD-L1 expression can be modulated by tumor cells. One feasible pathway is induced by IFNγ production and subsequent IFNGR/JAK/STAT signaling in tumor cells, which depends on TILs (72, 73). Thus, the correlation between PD-L1 overexpression and increased tumor-infiltrating T cells in sarcomas might indicate a higher capacity of sarcomas to evade immune surveillance. However, inhibitors targeting the PD1/PD-L1 axis have not resulted in satisfactory results in sarcomas. The combination of immunotherapy with other therapeutics is postulated to show improved responses over checkpoint inhibitor monotherapies (74, 75), and investigations to explore the underlying mechanisms by which PD-L1 is involved in sarcoma are encouraged.

Several limitations in our analysis should be acknowledged. First, there might be some potential sources of bias. The HRs and their corresponding 95% CIs were extracted indirectly or directly from the studies, including from multivariate and univariate analyses, which might lead to statistical bias. Moreover, most studies included in this meta-analysis were retrospective, investigating patients with osteosarcoma and STS. A limited number of studies of other rare sarcomas were included, which might lead to subject selection bias. In addition, some other underlying sources of publication bias should be considered, such as unpublished negative results (76) or studies that could not be included due to language limitations or insufficient data (77). Second, due to the limited number of studies included for some outcomes, the results should be interpreted with caution. Third, a lack of uniformity in the definitions of some clinicopathological outcomes was observed, such as tumor stage, size and depth, and patient age, which might be due to the authors' preferences or the characteristics of certain sarcomas, and the results should be interpreted with caution. In addition, although the onset of sarcomas is age dependent and the immune context might play a different role in young patients than in elderly patients, we could not obtain a conclusive result regarding whether PD-L1 expression was correlated with patient age due to the limited number of studies and the lack of uniformity in age classification. In addition, PD-L1 expression has been considered to correlate with tumor response to anti-PD-1/PD-L1 agents in melanoma patients (65), but no study investigated the correlation between PD-L1 expression and administration of PD-L1 inhibitors, which may be a very important factor for evaluating their efficacy. Because sufficient data could not be extracted, the correlation between PD-L1 expression and PD1-positive tumors was not assessed. Sarcomas are a group of rare malignancies with low morbidity, and there were not sufficient numbers of studies for some tumor subtypes; therefore, more investigations are encouraged to clarify the significance of PD-L1 for those rare tumor subtypes.

Moreover, the assessment of PD-L1 expression through IHC is advocated by many studies, and a variety of antibodies and cutoff values for positive PD-L1 labeling are used. Other assay methods, such as qPCR and RNA sequencing, are also used to assess PD-L1 expression. To avoid selection bias and missing important data, we included studies using IHC and other assay methods. The results of the subgroup analysis by assay method showed that high PD-L1 expression predicted poor OS at both the protein level (HR 1.36, 95% CI 1.00–1.86, *P* < 0.01) and the mRNA level (HR 2.06, 95% CI 1.42–2.98, *P* < 0.01), and the assay method was not an underlying source of heterogeneity. As different cutoff values for PD-L1 positivity might affect the reliability of the evaluation, a subgroup analysis by cutoff value for IHC data was conducted. The results showed that PD-L1 overexpression could not predict poor OS with a defined cutoff value of ≥1% of cells, ≥5% of cells, or ≥10% of cells (Supplementary Figure 15), suggesting that the cutoff value could impact the prognostic value of PD-L1. It has been reported that different IHC methodologies for PD-L1 assessment provide different results (78); therefore, other approaches should be employed to improve the evaluation of PD-L1 expression in clinical practice (79), including in studies of patients with sarcomas.

## Conclusions

In this meta-analysis, the results showed that PD-L1 overexpression could predict poor survival and was correlated with adverse tumor status in sarcoma patients. The results suggest that PD-L1 is a valuable prognostic biomarker in bone and soft tissue sarcomas, although more well-designed prospective studies with appropriate multivariate analyses are needed to validate our results.

## Data Availability Statement

All datasets analyzed for this study are included in the article/Supplementary Material.

## Author Contributions

FW and ZZ conceived and designed the project. FW, TY, and HZ analyzed and interpreted the data. FW, TY, and ZZ wrote the paper. All the authors have read and approved the final version of the manuscript and participated in acquiring the data.

## Conflict of Interest

The authors declare that the research was conducted in the absence of any commercial or financial relationships that could be construed as a potential conflict of interest.
