# Supplementary material for: Prognostic Value of Programmed Cell Death 1 Ligand-1 in Patients With Bone and Soft Tissue Sarcomas: A Systemic and Comprehensive Meta-Analysis Based on 3,680 Patients
Source: Front Oncol. 2020 Jun 2;10:749. doi: 10.3389/fonc.2020.00749 (PMC7280448; doi:10.3389/fonc.2020.00749)
Supplement: Supplementary file 1 [file Presentation_1.PPTX]

## Slide 1
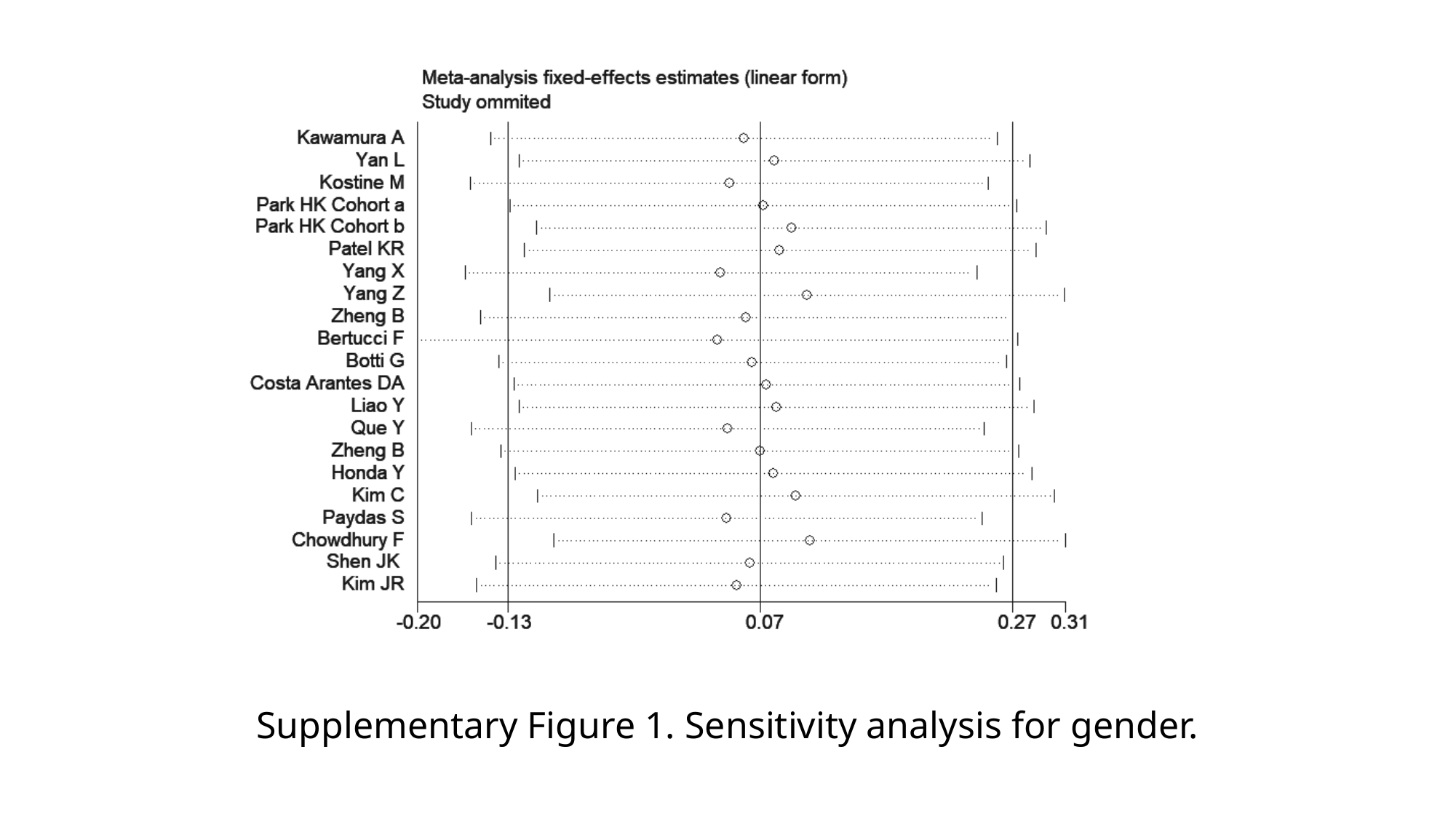

Supplementary Figure 1. Sensitivity analysis for gender.

## Slide 2
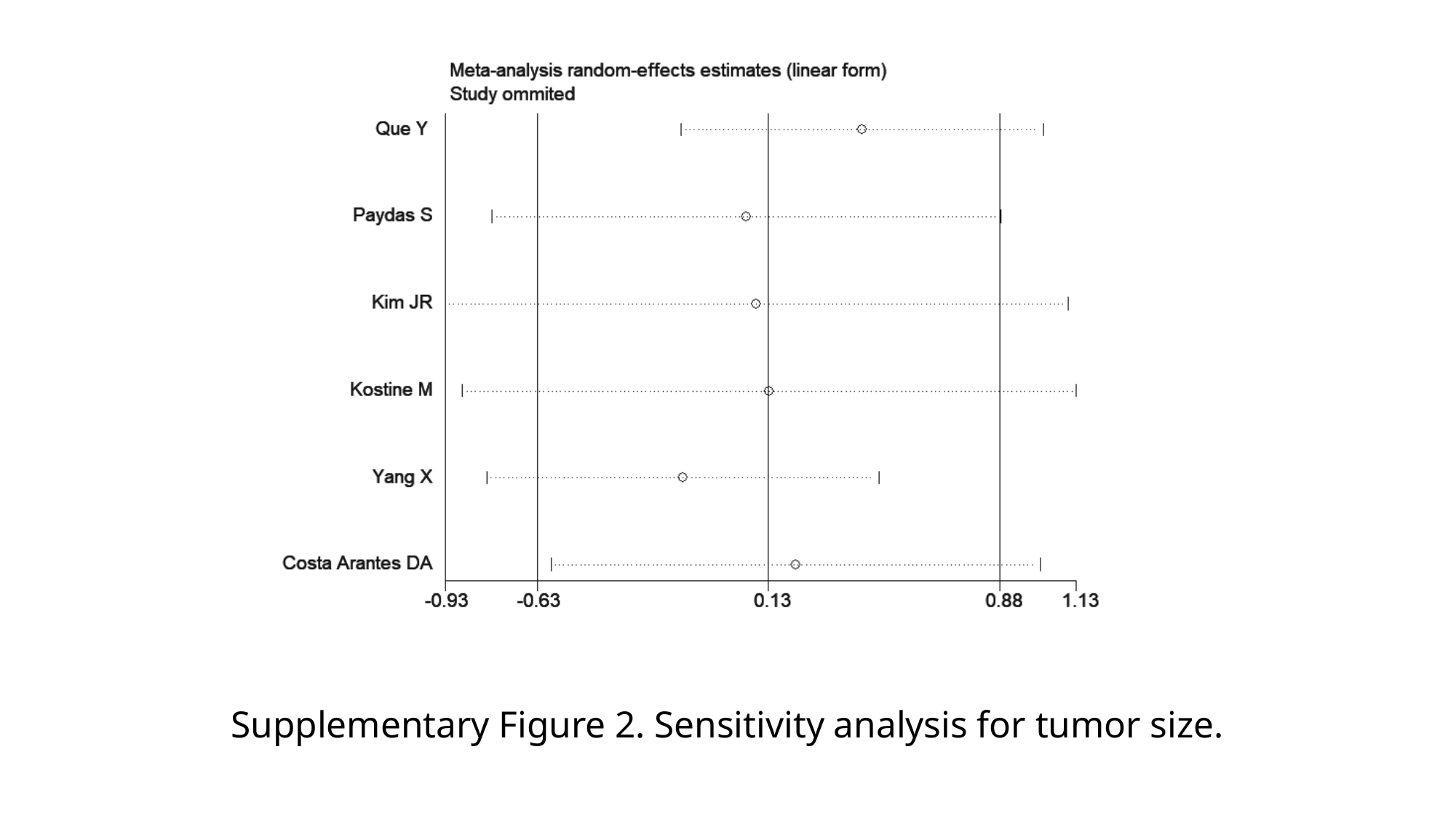

Supplementary Figure 2. Sensitivity analysis for tumor size.

## Slide 3
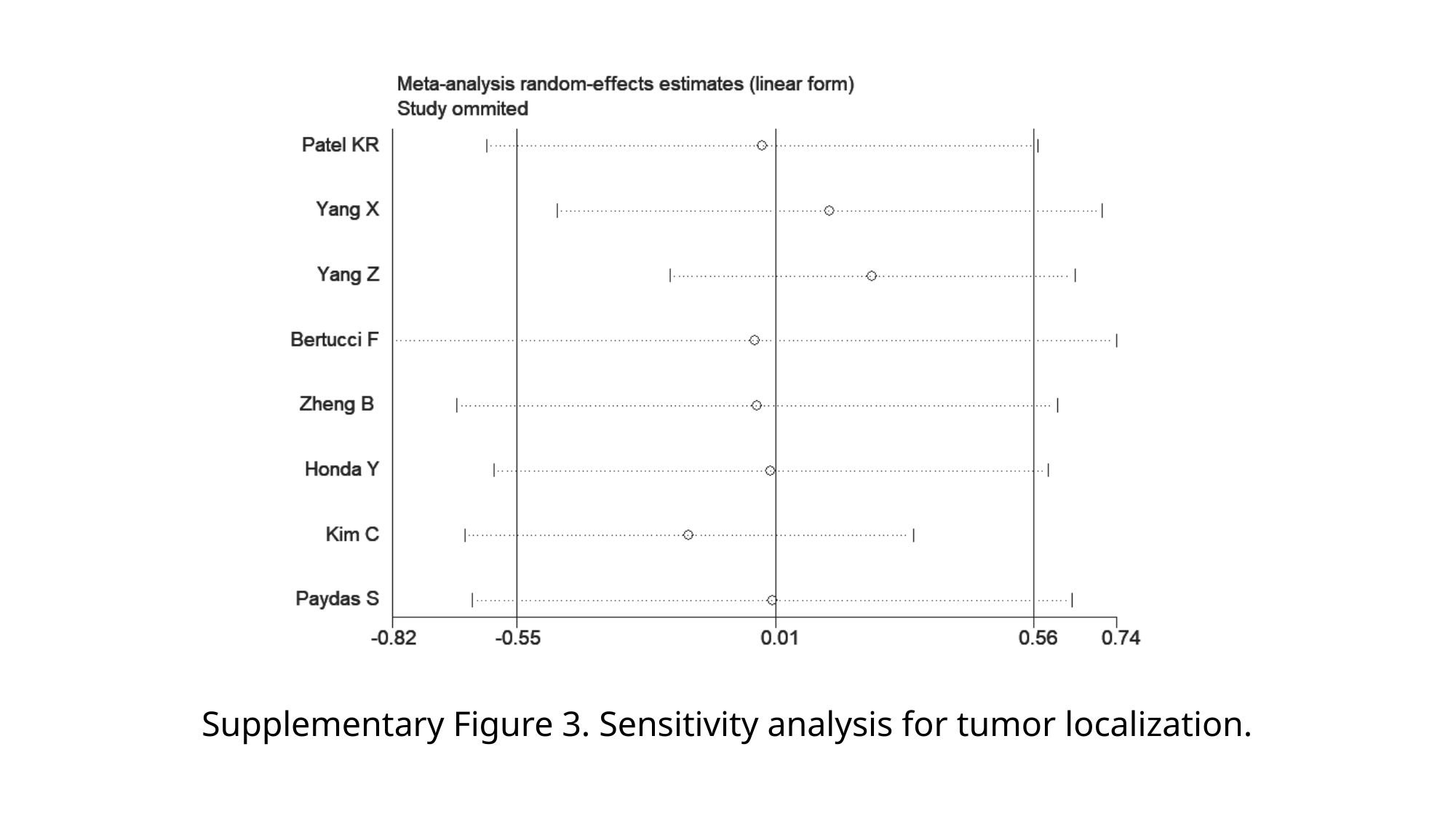

Supplementary Figure 3. Sensitivity analysis for tumor localization.

## Slide 4
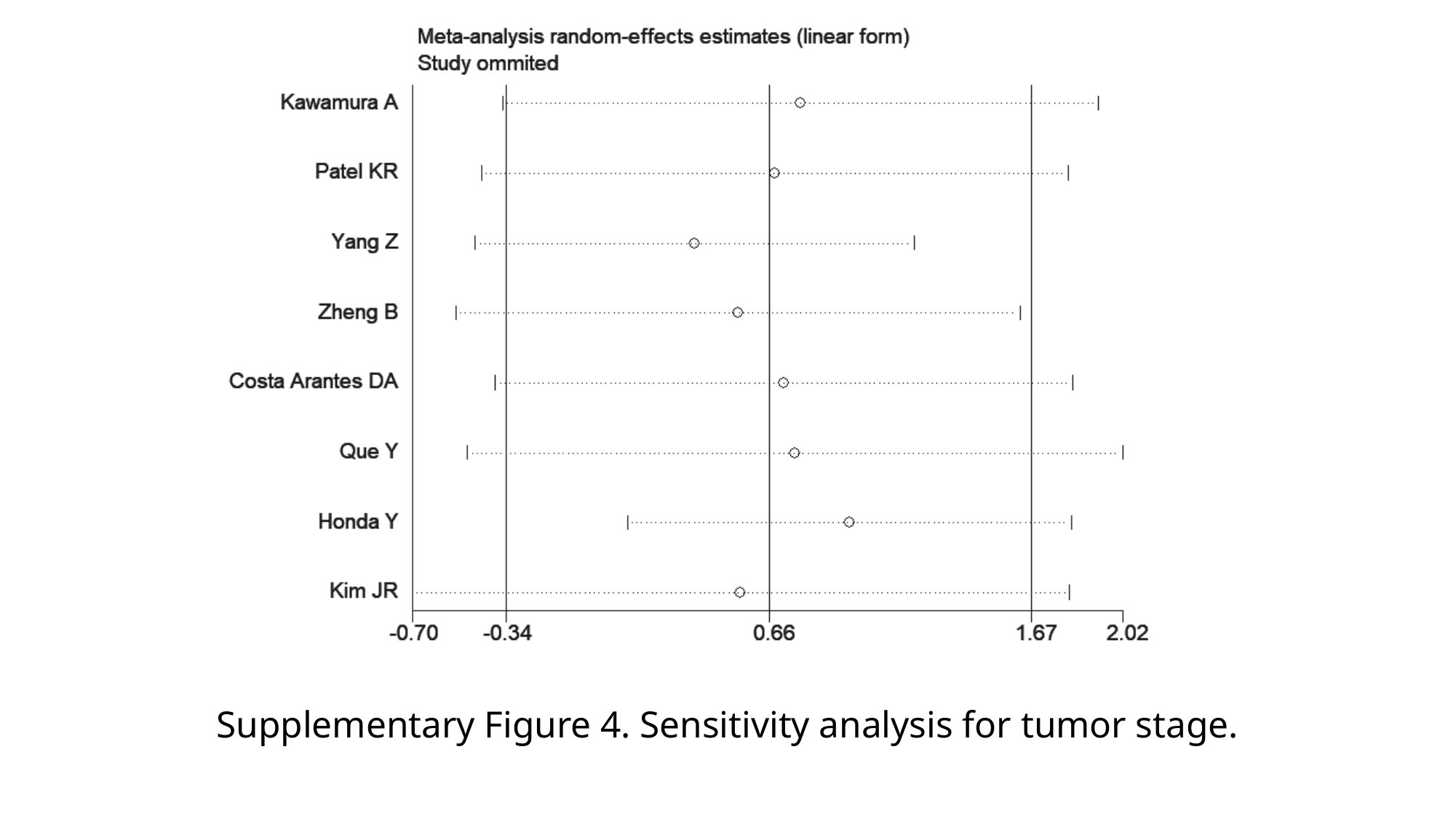

Supplementary Figure 4. Sensitivity analysis for tumor stage.

## Slide 5
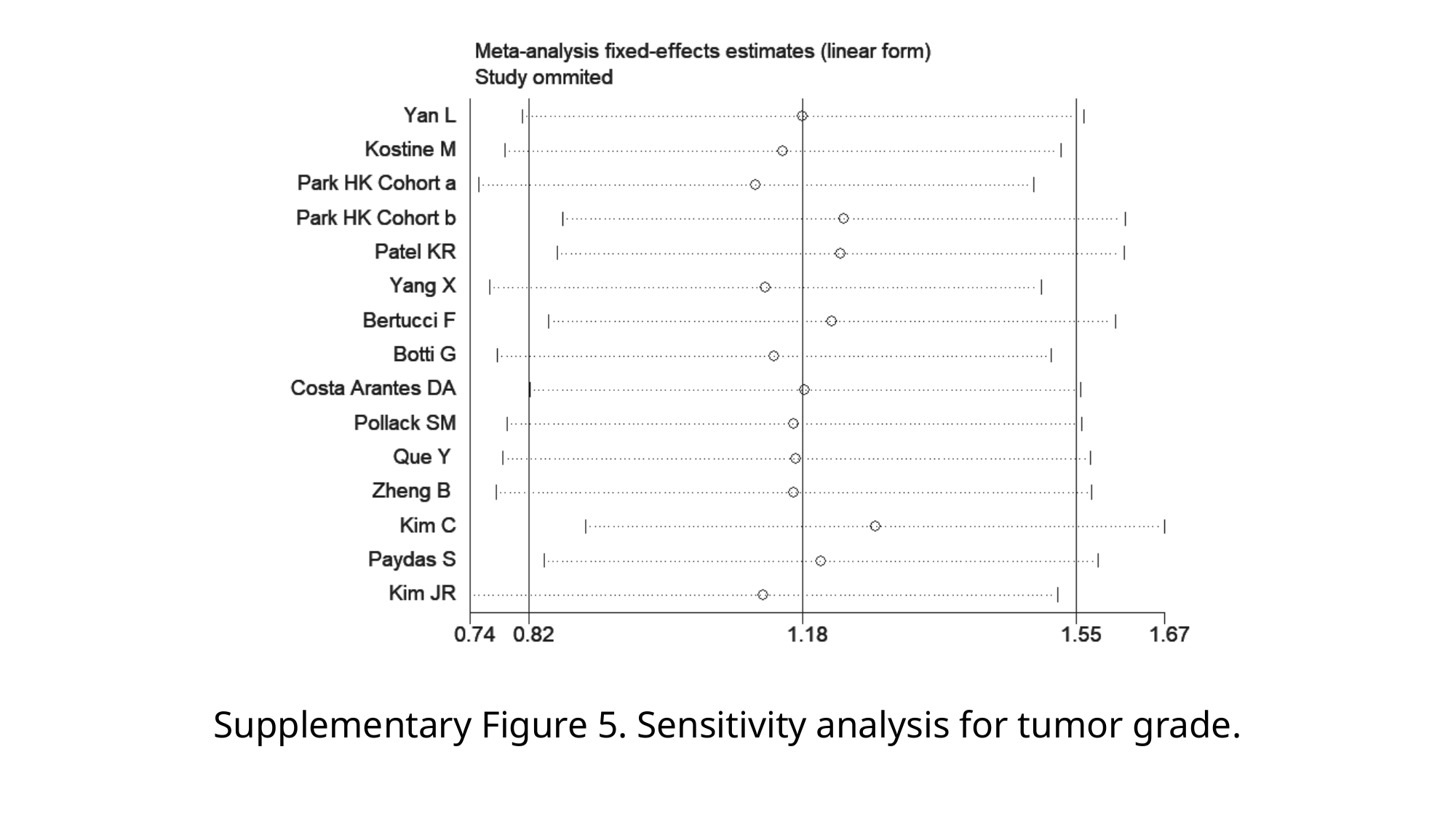

Supplementary Figure 5. Sensitivity analysis for tumor grade.

## Slide 6
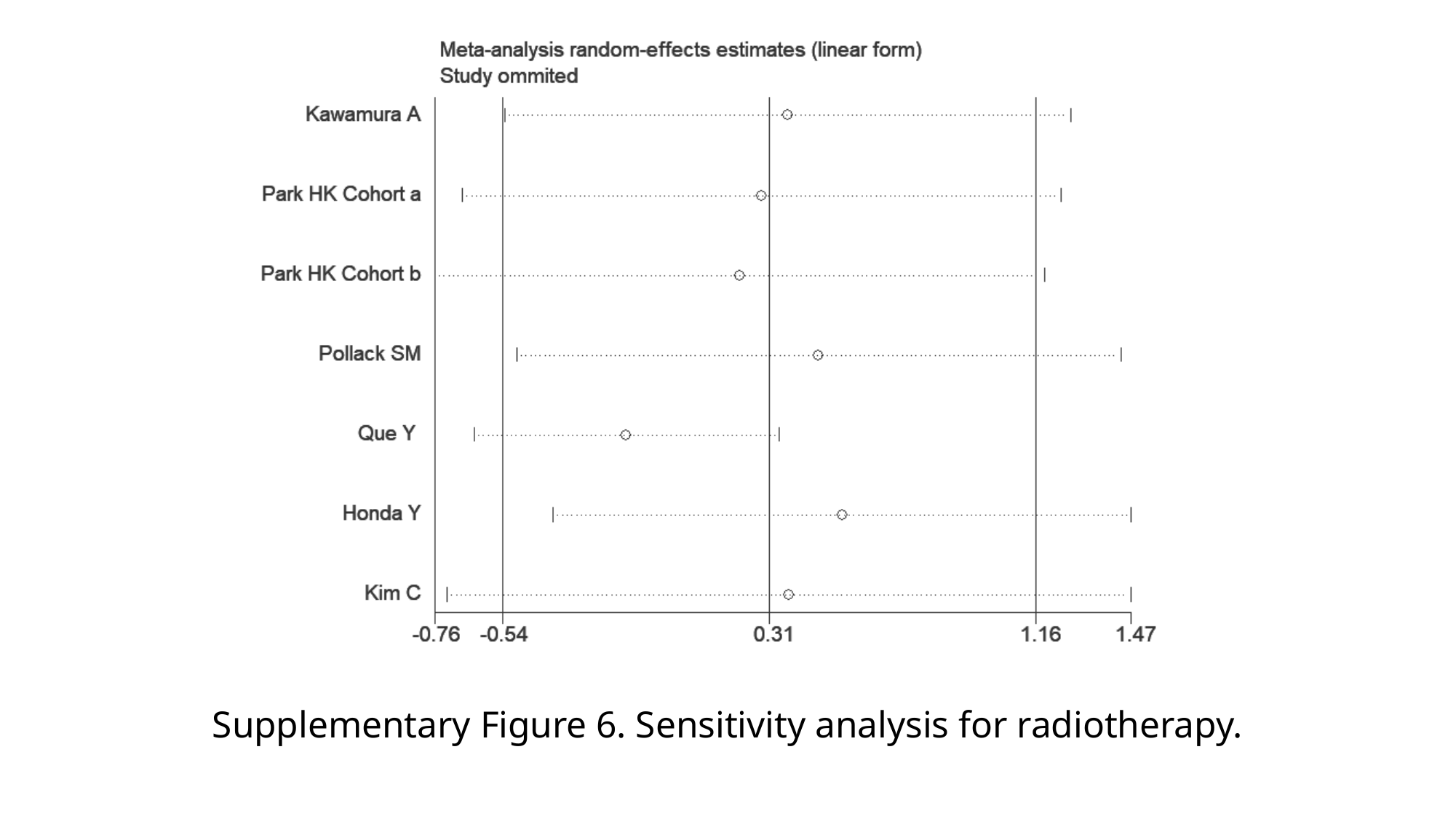

Supplementary Figure 6. Sensitivity analysis for radiotherapy.

## Slide 7
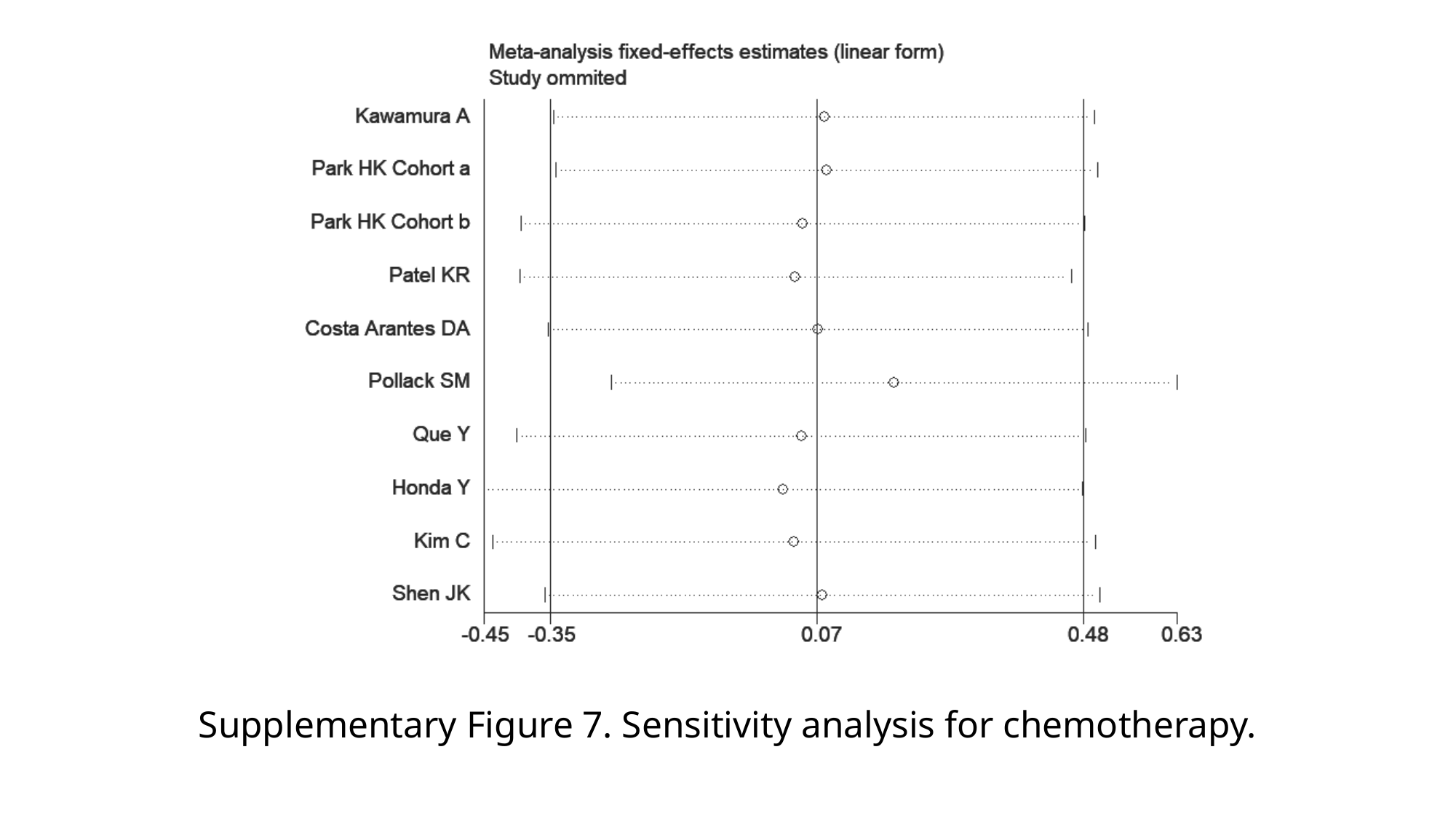

Supplementary Figure 7. Sensitivity analysis for chemotherapy.

## Slide 8
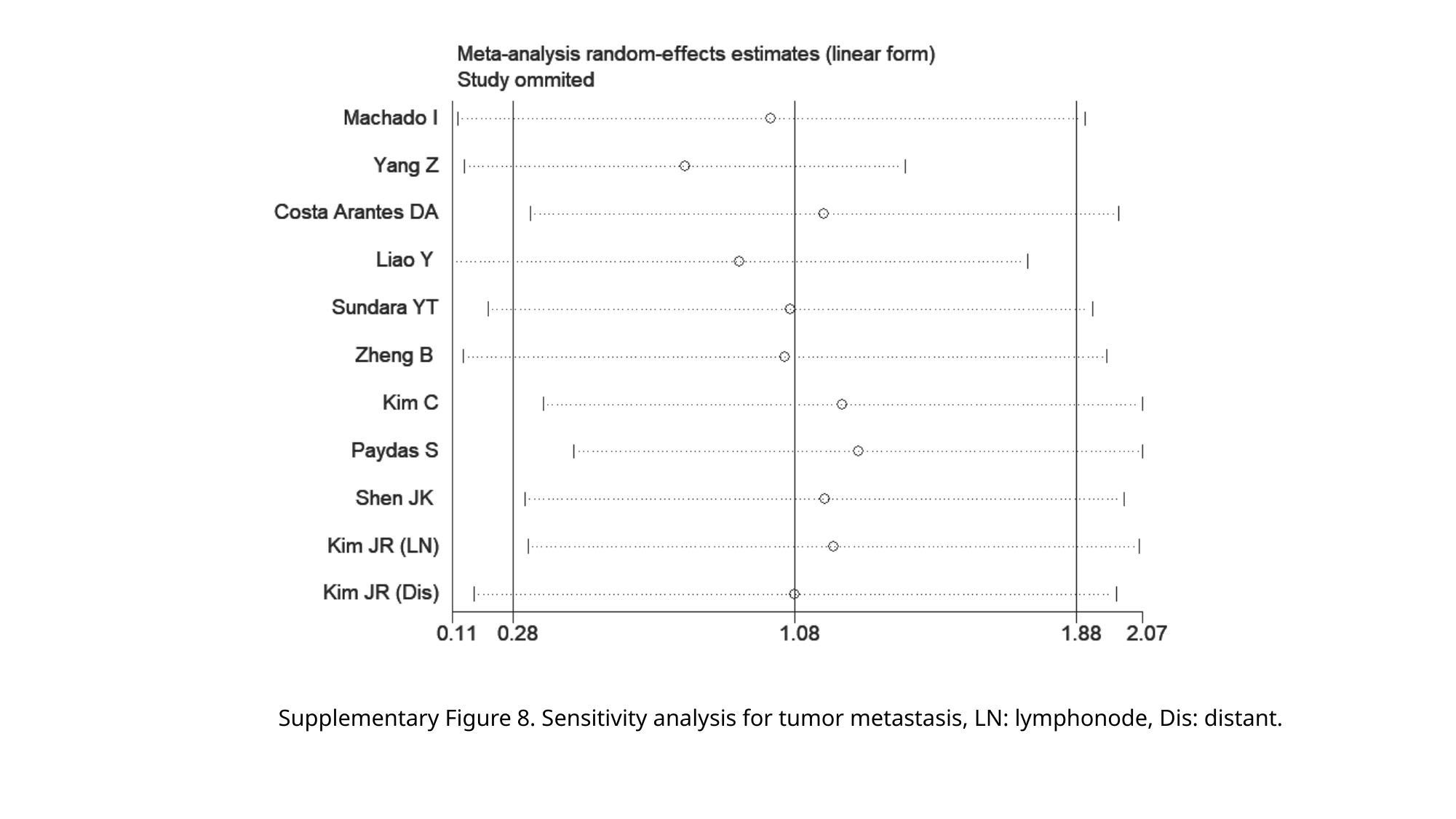

Supplementary Figure 8. Sensitivity analysis for tumor metastasis, LN: lymphonode, Dis: distant.

## Slide 9
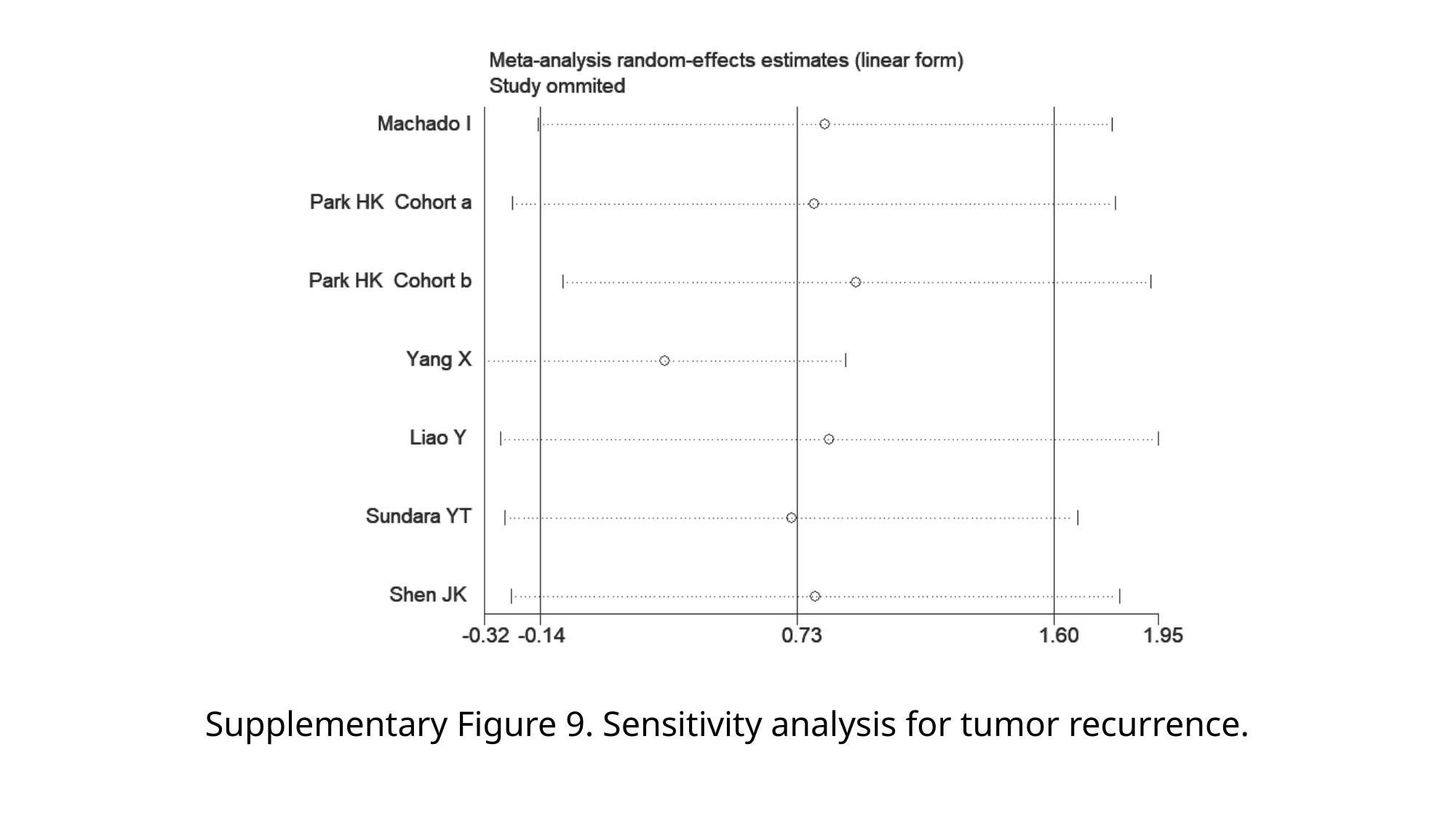

Supplementary Figure 9. Sensitivity analysis for tumor recurrence.

## Slide 10
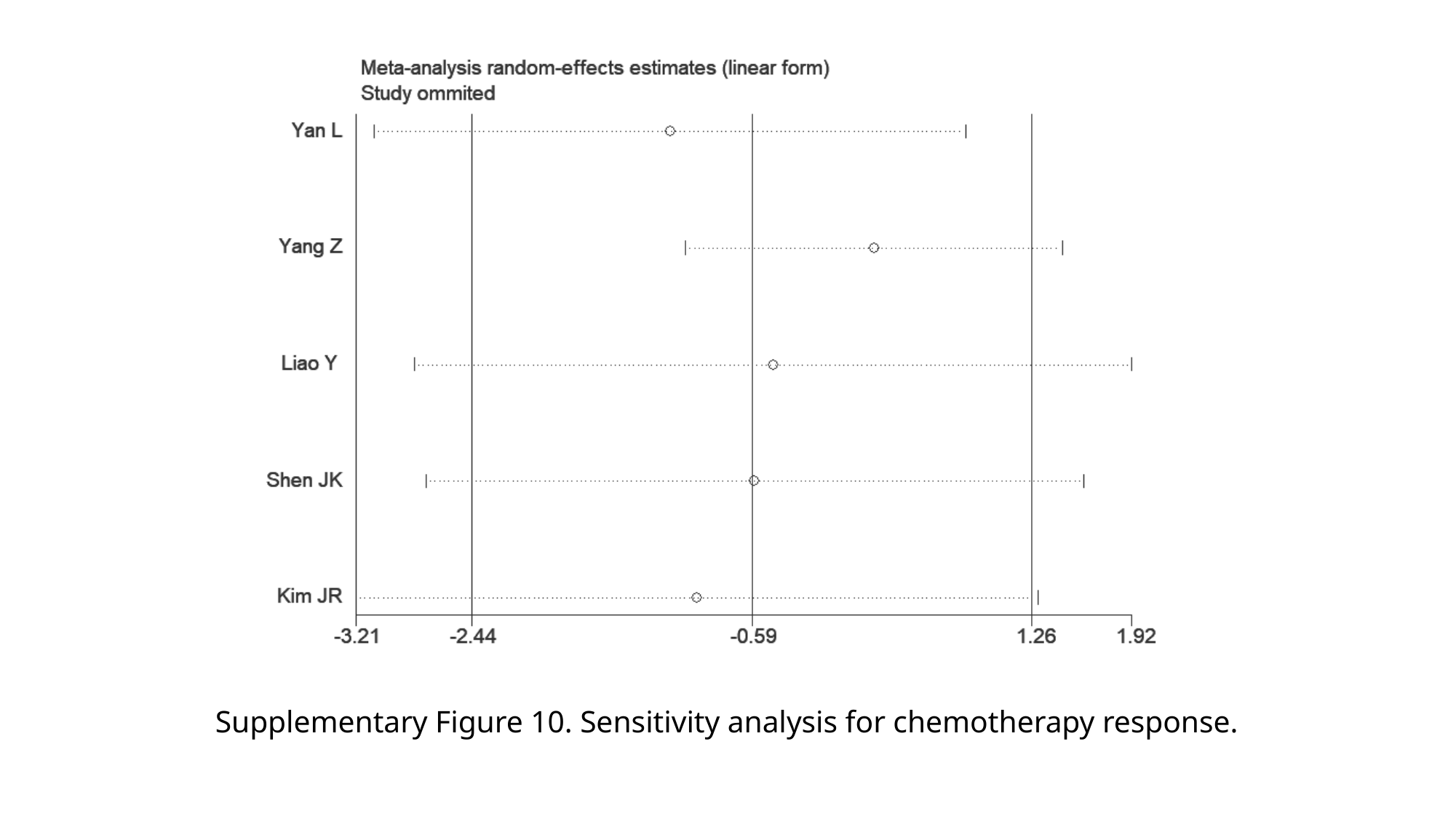

Supplementary Figure 10. Sensitivity analysis for chemotherapy response.

## Slide 11
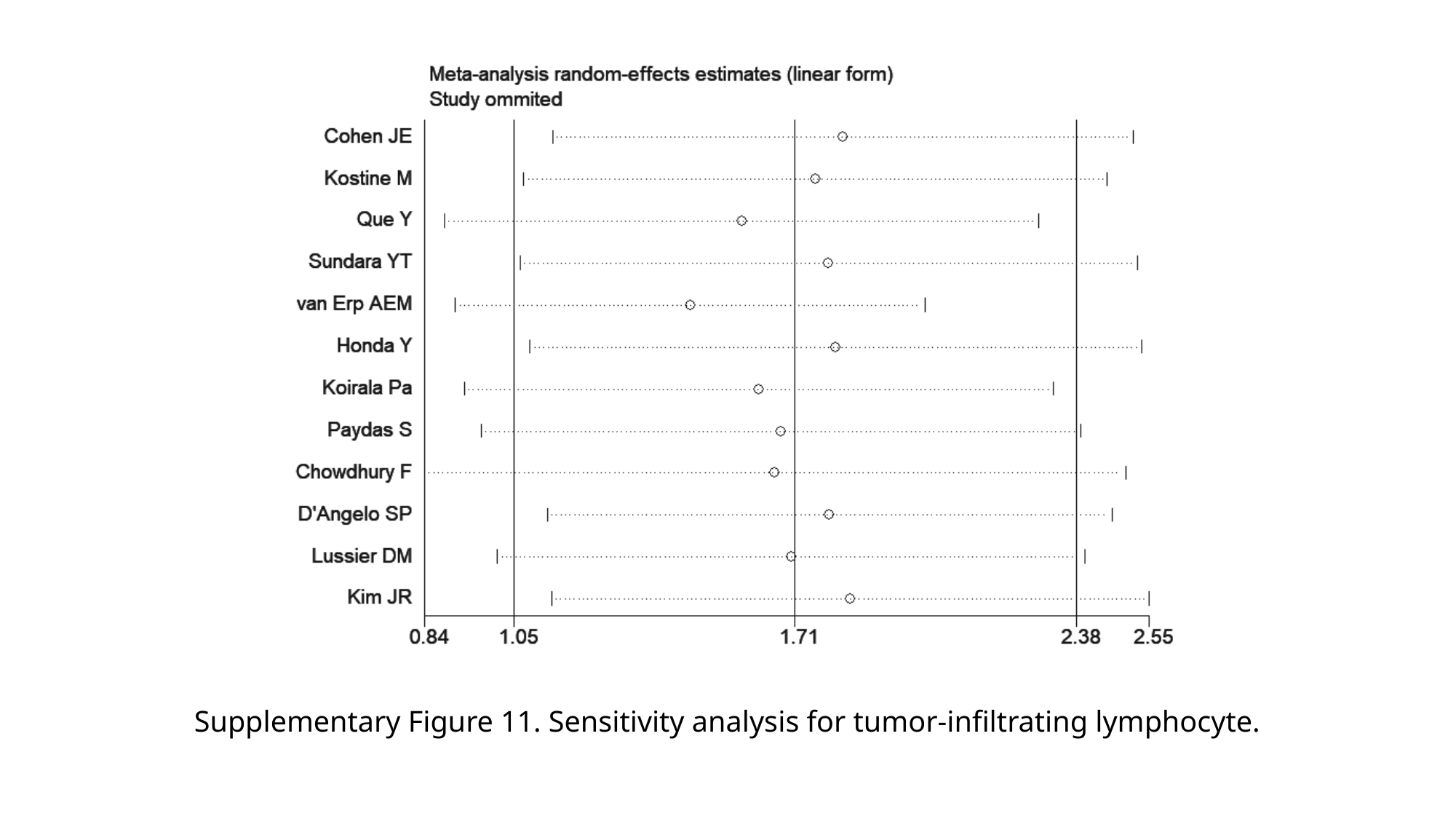

Supplementary Figure 11. Sensitivity analysis for tumor-infiltrating lymphocyte.

## Slide 12
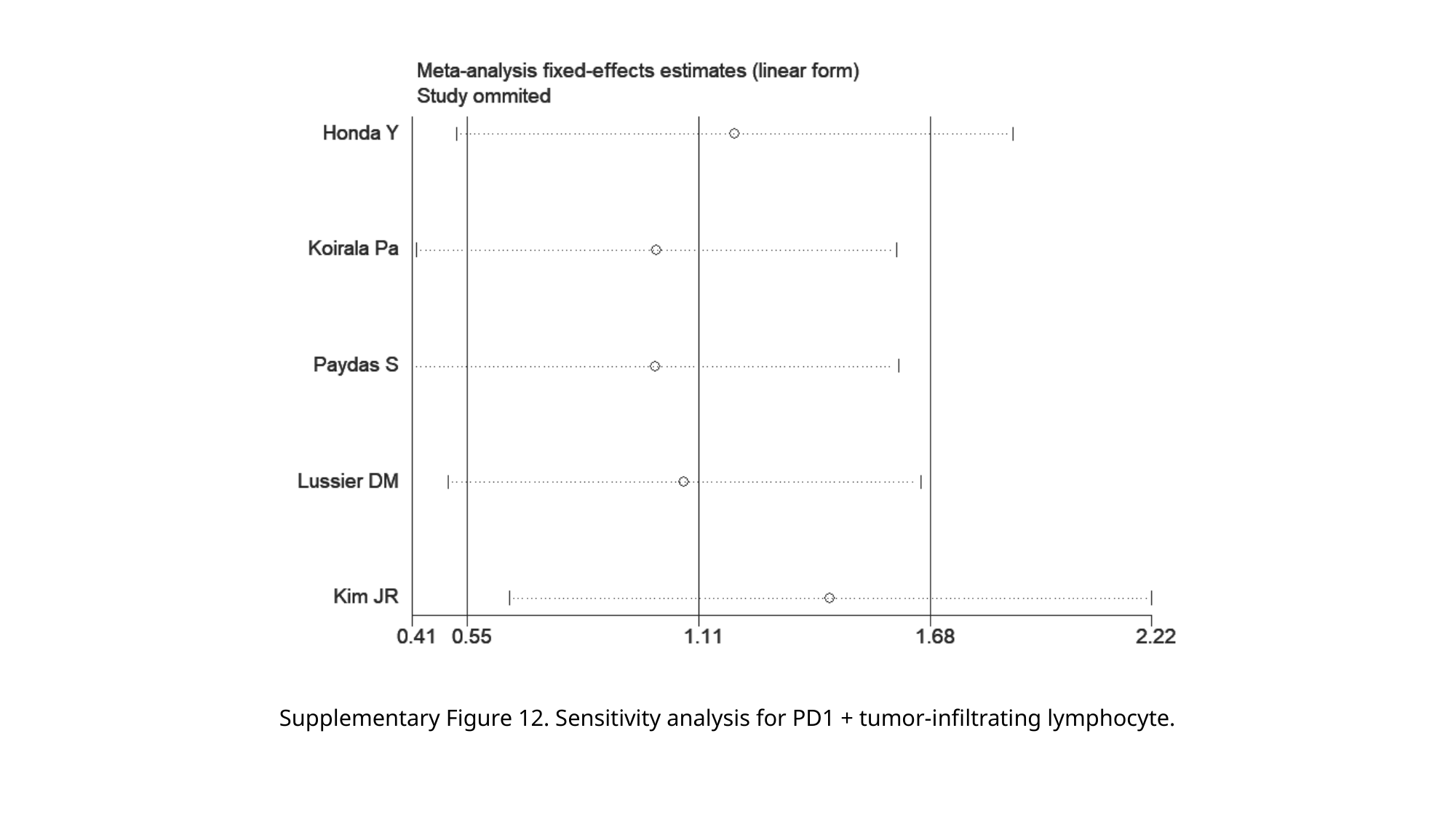

Supplementary Figure 12. Sensitivity analysis for PD1 + tumor-infiltrating lymphocyte.

## Slide 13
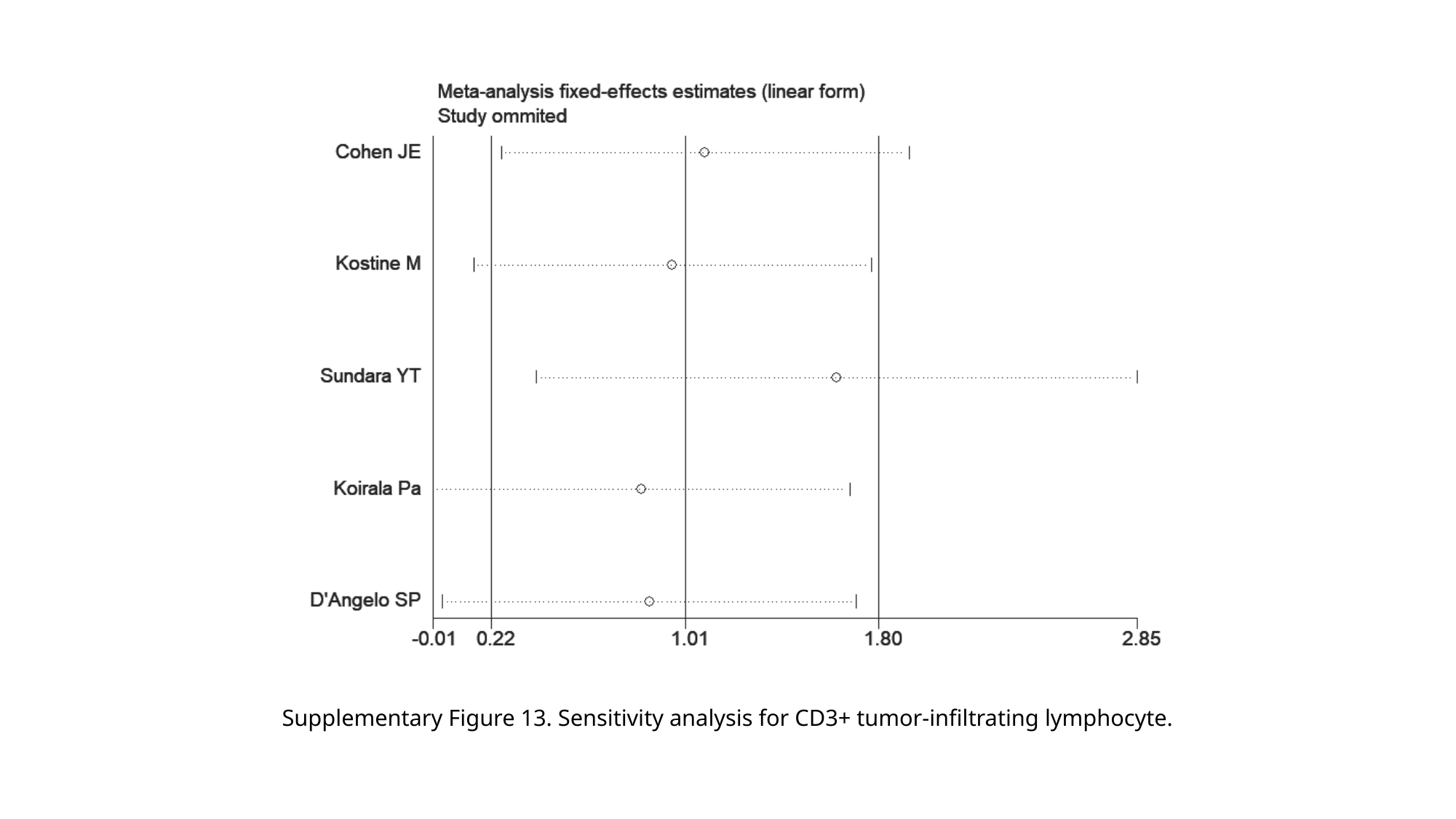

Supplementary Figure 13. Sensitivity analysis for CD3+ tumor-infiltrating lymphocyte.

## Slide 14
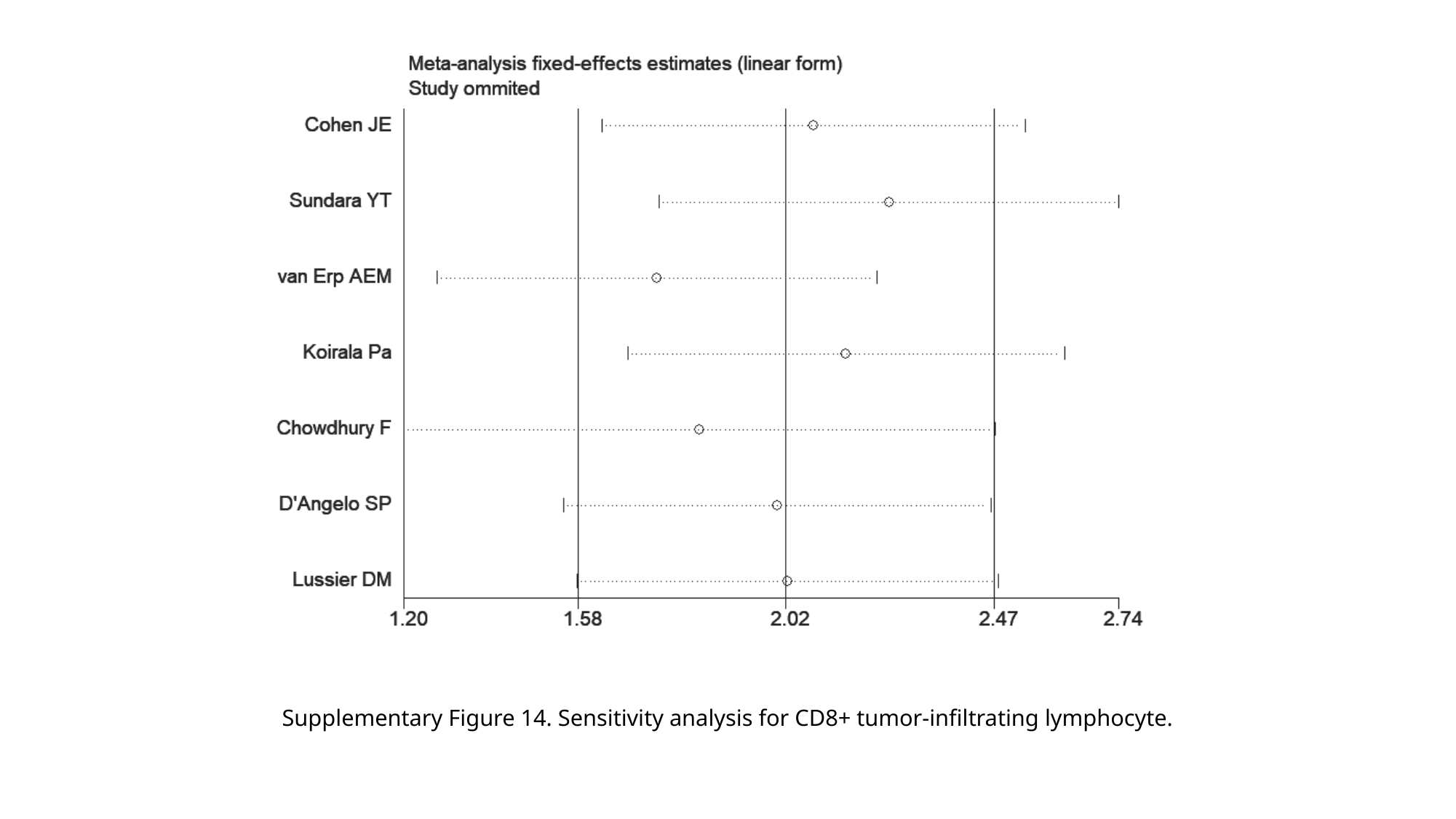

Supplementary Figure 14. Sensitivity analysis for CD8+ tumor-infiltrating lymphocyte.

## Slide 15
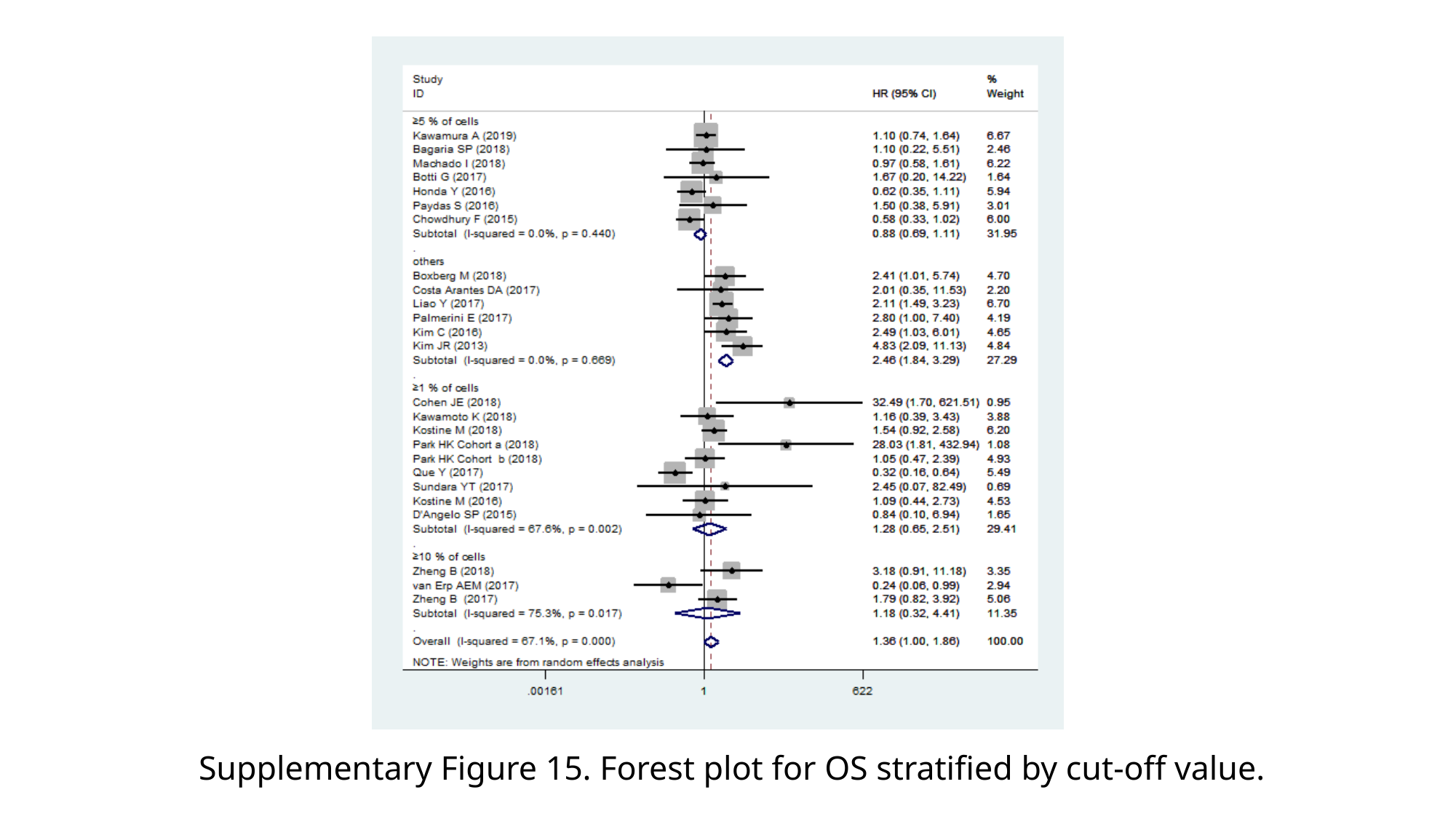

Supplementary Figure 15. Forest plot for OS stratified by cut-off value.

## Slide 16
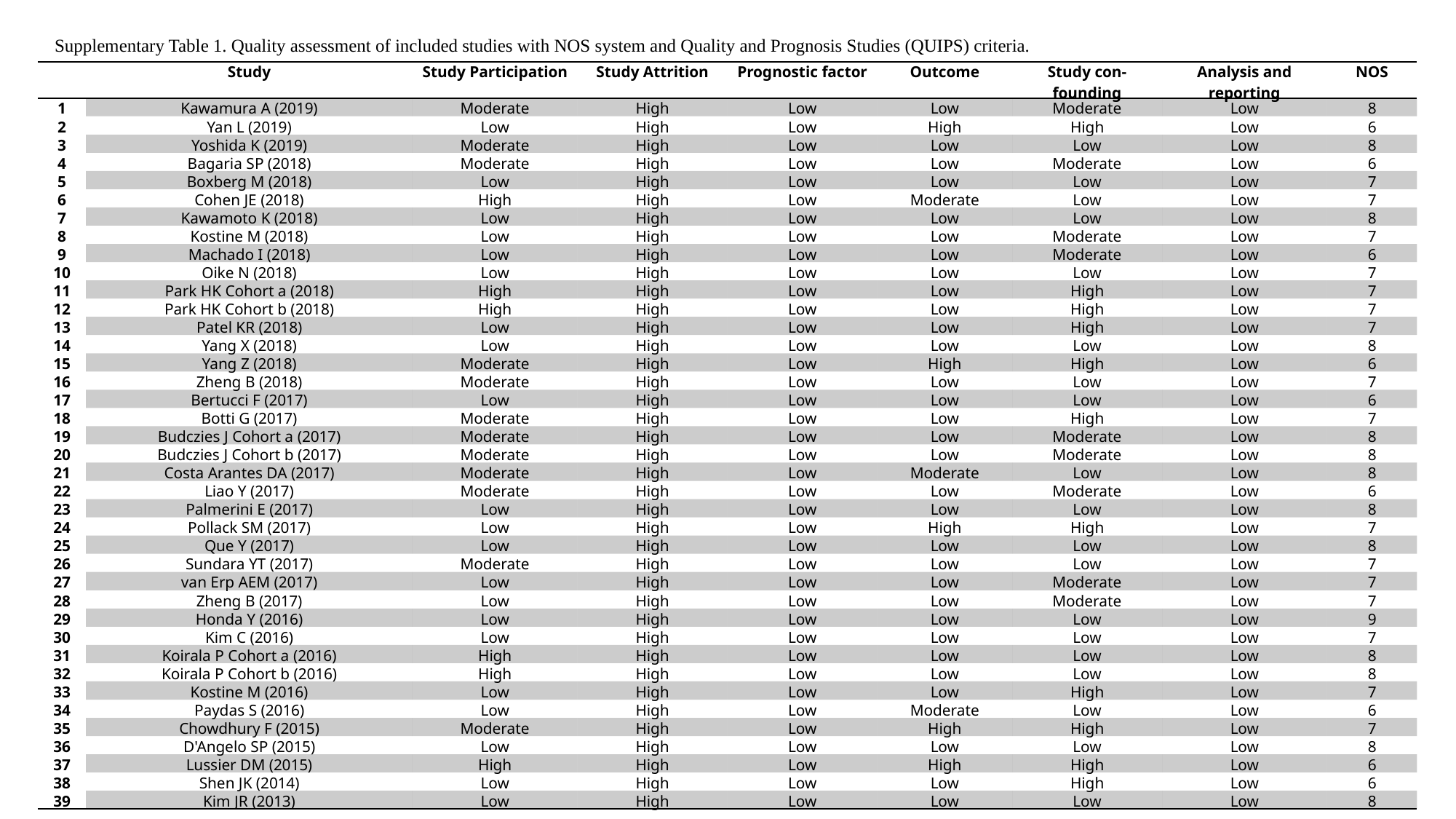

Supplementary Table 1. Quality assessment of included studies with NOS system and Quality and Prognosis Studies (QUIPS) criteria.
| | Study | Study Participation | Study Attrition | Prognostic factor | Outcome | Study con-founding | Analysis and reporting | NOS |
| --- | --- | --- | --- | --- | --- | --- | --- | --- |
| 1 | Kawamura A (2019) | Moderate | High | Low | Low | Moderate | Low | 8 |
| 2 | Yan L (2019) | Low | High | Low | High | High | Low | 6 |
| 3 | Yoshida K (2019) | Moderate | High | Low | Low | Low | Low | 8 |
| 4 | Bagaria SP (2018) | Moderate | High | Low | Low | Moderate | Low | 6 |
| 5 | Boxberg M (2018) | Low | High | Low | Low | Low | Low | 7 |
| 6 | Cohen JE (2018) | High | High | Low | Moderate | Low | Low | 7 |
| 7 | Kawamoto K (2018) | Low | High | Low | Low | Low | Low | 8 |
| 8 | Kostine M (2018) | Low | High | Low | Low | Moderate | Low | 7 |
| 9 | Machado I (2018) | Low | High | Low | Low | Moderate | Low | 6 |
| 10 | Oike N (2018) | Low | High | Low | Low | Low | Low | 7 |
| 11 | Park HK Cohort a (2018) | High | High | Low | Low | High | Low | 7 |
| 12 | Park HK Cohort b (2018) | High | High | Low | Low | High | Low | 7 |
| 13 | Patel KR (2018) | Low | High | Low | Low | High | Low | 7 |
| 14 | Yang X (2018) | Low | High | Low | Low | Low | Low | 8 |
| 15 | Yang Z (2018) | Moderate | High | Low | High | High | Low | 6 |
| 16 | Zheng B (2018) | Moderate | High | Low | Low | Low | Low | 7 |
| 17 | Bertucci F (2017) | Low | High | Low | Low | Low | Low | 6 |
| 18 | Botti G (2017) | Moderate | High | Low | Low | High | Low | 7 |
| 19 | Budczies J Cohort a (2017) | Moderate | High | Low | Low | Moderate | Low | 8 |
| 20 | Budczies J Cohort b (2017) | Moderate | High | Low | Low | Moderate | Low | 8 |
| 21 | Costa Arantes DA (2017) | Moderate | High | Low | Moderate | Low | Low | 8 |
| 22 | Liao Y (2017) | Moderate | High | Low | Low | Moderate | Low | 6 |
| 23 | Palmerini E (2017) | Low | High | Low | Low | Low | Low | 8 |
| 24 | Pollack SM (2017) | Low | High | Low | High | High | Low | 7 |
| 25 | Que Y (2017) | Low | High | Low | Low | Low | Low | 8 |
| 26 | Sundara YT (2017) | Moderate | High | Low | Low | Low | Low | 7 |
| 27 | van Erp AEM (2017) | Low | High | Low | Low | Moderate | Low | 7 |
| 28 | Zheng B (2017) | Low | High | Low | Low | Moderate | Low | 7 |
| 29 | Honda Y (2016) | Low | High | Low | Low | Low | Low | 9 |
| 30 | Kim C (2016) | Low | High | Low | Low | Low | Low | 7 |
| 31 | Koirala P Cohort a (2016) | High | High | Low | Low | Low | Low | 8 |
| 32 | Koirala P Cohort b (2016) | High | High | Low | Low | Low | Low | 8 |
| 33 | Kostine M (2016) | Low | High | Low | Low | High | Low | 7 |
| 34 | Paydas S (2016) | Low | High | Low | Moderate | Low | Low | 6 |
| 35 | Chowdhury F (2015) | Moderate | High | Low | High | High | Low | 7 |
| 36 | D'Angelo SP (2015) | Low | High | Low | Low | Low | Low | 8 |
| 37 | Lussier DM (2015) | High | High | Low | High | High | Low | 6 |
| 38 | Shen JK (2014) | Low | High | Low | Low | High | Low | 6 |
| 39 | Kim JR (2013) | Low | High | Low | Low | Low | Low | 8 |

## Slide 17
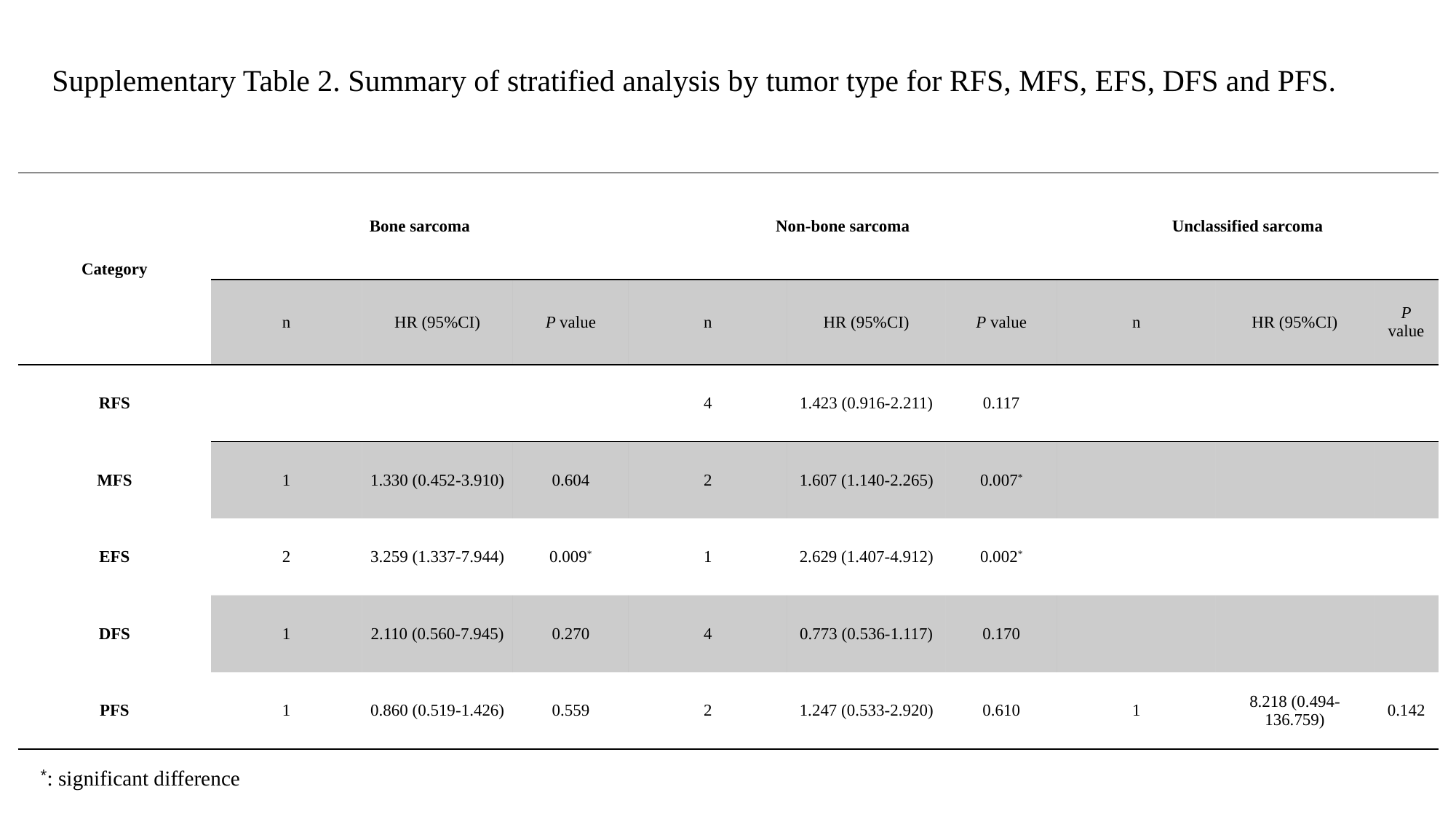

# Supplementary Table 2. Summary of stratified analysis by tumor type for RFS, MFS, EFS, DFS and PFS.
| Category | Bone sarcoma | | | Non-bone sarcoma | | | Unclassified sarcoma | | |
| --- | --- | --- | --- | --- | --- | --- | --- | --- | --- |
| | n | HR (95%CI) | P value | n | HR (95%CI) | P value | n | HR (95%CI) | P value |
| RFS | | | | 4 | 1.423 (0.916-2.211) | 0.117 | | | |
| MFS | 1 | 1.330 (0.452-3.910) | 0.604 | 2 | 1.607 (1.140-2.265) | 0.007\* | | | |
| EFS | 2 | 3.259 (1.337-7.944) | 0.009\* | 1 | 2.629 (1.407-4.912) | 0.002\* | | | |
| DFS | 1 | 2.110 (0.560-7.945) | 0.270 | 4 | 0.773 (0.536-1.117) | 0.170 | | | |
| PFS | 1 | 0.860 (0.519-1.426) | 0.559 | 2 | 1.247 (0.533-2.920) | 0.610 | 1 | 8.218 (0.494-136.759) | 0.142 |
*: significant difference

## Slide 18
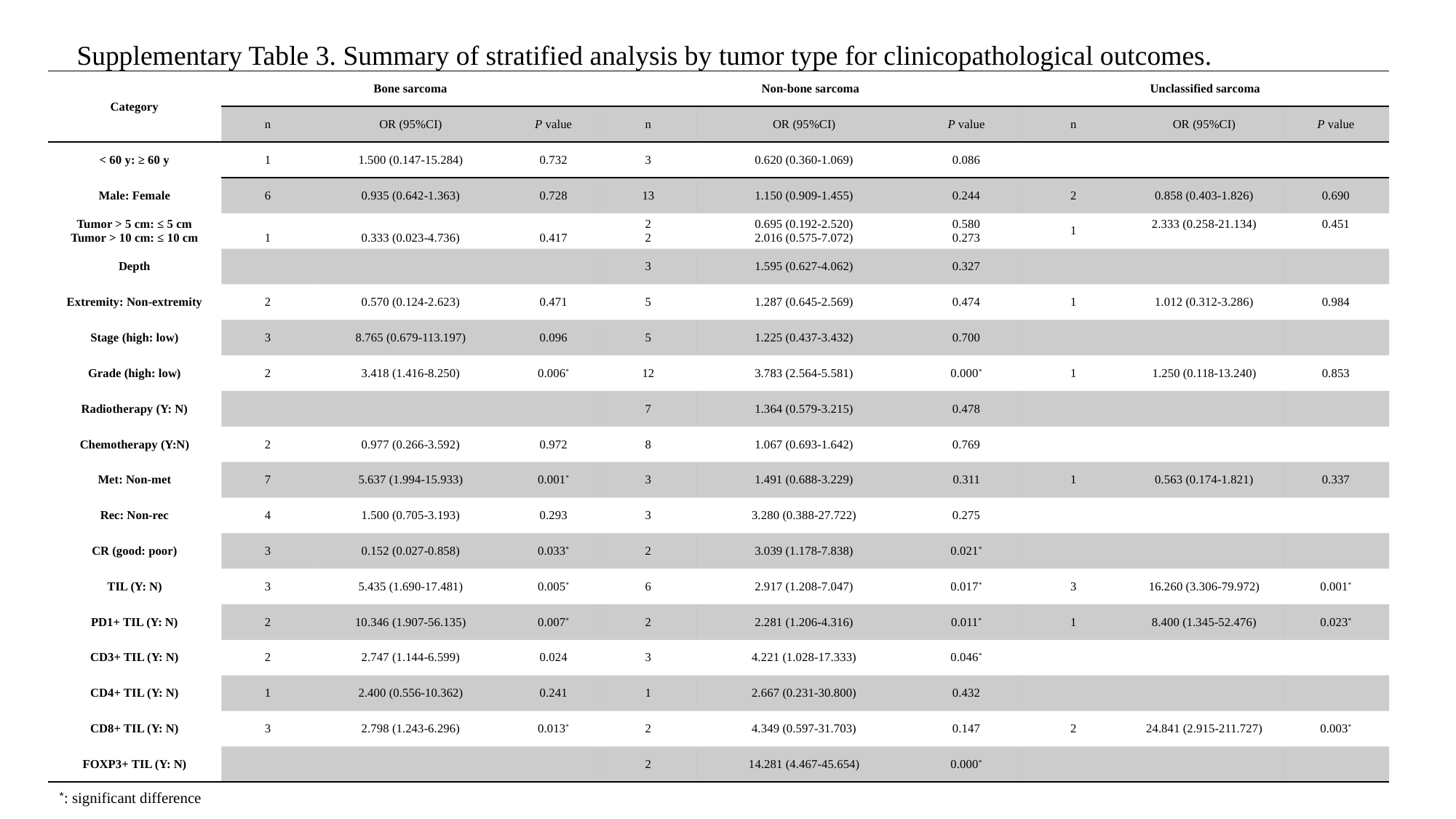

Supplementary Table 3. Summary of stratified analysis by tumor type for clinicopathological outcomes.
| Category | Bone sarcoma | | | Non-bone sarcoma | | | Unclassified sarcoma | | |
| --- | --- | --- | --- | --- | --- | --- | --- | --- | --- |
| | n | OR (95%CI) | P value | n | OR (95%CI) | P value | n | OR (95%CI) | P value |
| < 60 y: ≥ 60 y | 1 | 1.500 (0.147-15.284) | 0.732 | 3 | 0.620 (0.360-1.069) | 0.086 | | | |
| Male: Female | 6 | 0.935 (0.642-1.363) | 0.728 | 13 | 1.150 (0.909-1.455) | 0.244 | 2 | 0.858 (0.403-1.826) | 0.690 |
| Tumor > 5 cm: ≤ 5 cm Tumor > 10 cm: ≤ 10 cm | 1 | 0.333 (0.023-4.736) | 0.417 | 2 2 | 0.695 (0.192-2.520) 2.016 (0.575-7.072) | 0.580 0.273 | 1 | 2.333 (0.258-21.134) | 0.451 |
| Depth | | | | 3 | 1.595 (0.627-4.062) | 0.327 | | | |
| Extremity: Non-extremity | 2 | 0.570 (0.124-2.623) | 0.471 | 5 | 1.287 (0.645-2.569) | 0.474 | 1 | 1.012 (0.312-3.286) | 0.984 |
| Stage (high: low) | 3 | 8.765 (0.679-113.197) | 0.096 | 5 | 1.225 (0.437-3.432) | 0.700 | | | |
| Grade (high: low) | 2 | 3.418 (1.416-8.250) | 0.006\* | 12 | 3.783 (2.564-5.581) | 0.000\* | 1 | 1.250 (0.118-13.240) | 0.853 |
| Radiotherapy (Y: N) | | | | 7 | 1.364 (0.579-3.215) | 0.478 | | | |
| Chemotherapy (Y:N) | 2 | 0.977 (0.266-3.592) | 0.972 | 8 | 1.067 (0.693-1.642) | 0.769 | | | |
| Met: Non-met | 7 | 5.637 (1.994-15.933) | 0.001\* | 3 | 1.491 (0.688-3.229) | 0.311 | 1 | 0.563 (0.174-1.821) | 0.337 |
| Rec: Non-rec | 4 | 1.500 (0.705-3.193) | 0.293 | 3 | 3.280 (0.388-27.722) | 0.275 | | | |
| CR (good: poor) | 3 | 0.152 (0.027-0.858) | 0.033\* | 2 | 3.039 (1.178-7.838) | 0.021\* | | | |
| TIL (Y: N) | 3 | 5.435 (1.690-17.481) | 0.005\* | 6 | 2.917 (1.208-7.047) | 0.017\* | 3 | 16.260 (3.306-79.972) | 0.001\* |
| PD1+ TIL (Y: N) | 2 | 10.346 (1.907-56.135) | 0.007\* | 2 | 2.281 (1.206-4.316) | 0.011\* | 1 | 8.400 (1.345-52.476) | 0.023\* |
| CD3+ TIL (Y: N) | 2 | 2.747 (1.144-6.599) | 0.024 | 3 | 4.221 (1.028-17.333) | 0.046\* | | | |
| CD4+ TIL (Y: N) | 1 | 2.400 (0.556-10.362) | 0.241 | 1 | 2.667 (0.231-30.800) | 0.432 | | | |
| CD8+ TIL (Y: N) | 3 | 2.798 (1.243-6.296) | 0.013\* | 2 | 4.349 (0.597-31.703) | 0.147 | 2 | 24.841 (2.915-211.727) | 0.003\* |
| FOXP3+ TIL (Y: N) | | | | 2 | 14.281 (4.467-45.654) | 0.000\* | | | |
*: significant difference

## Slide 19
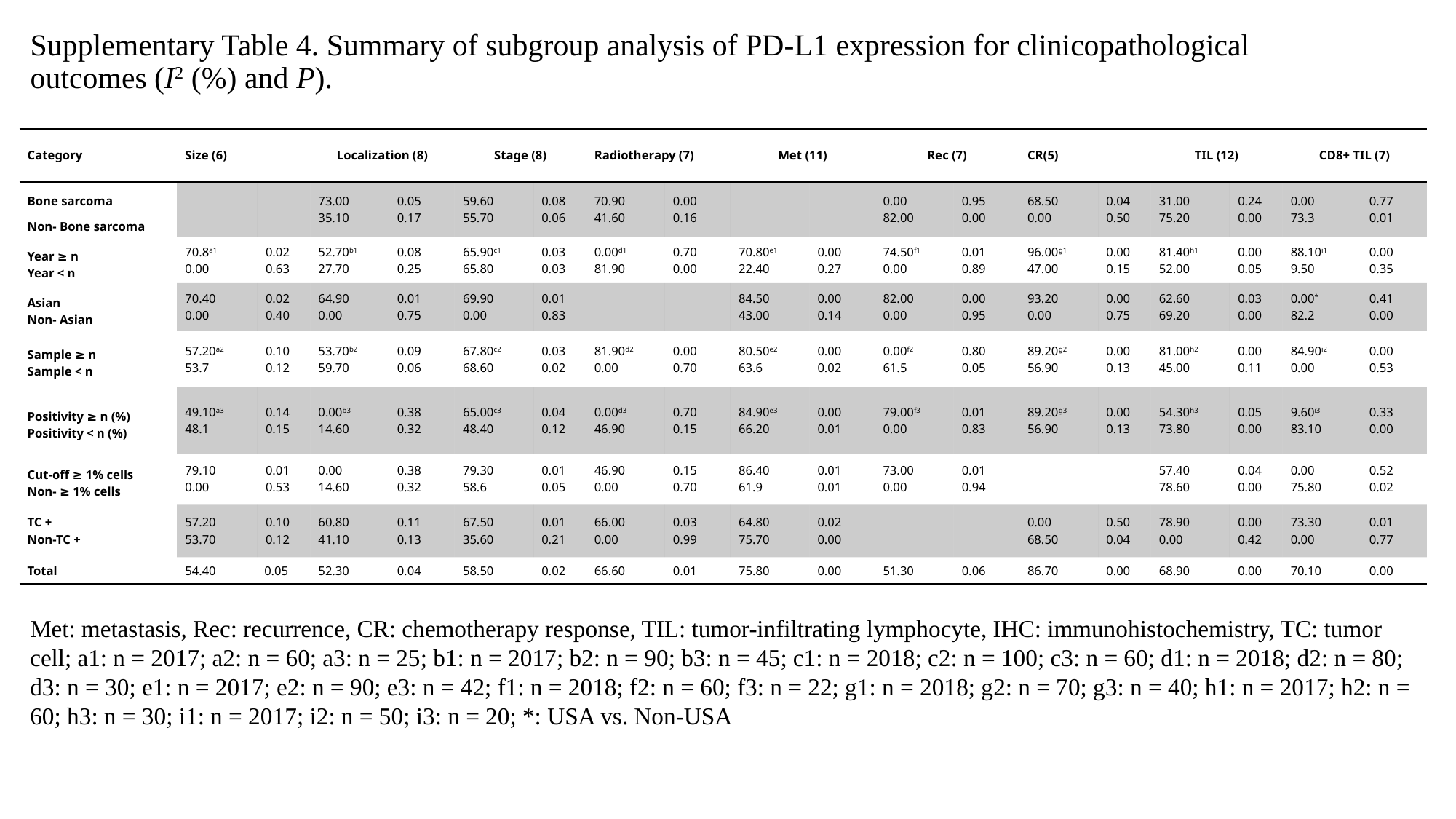

# Supplementary Table 4. Summary of subgroup analysis of PD-L1 expression for clinicopathological outcomes (I2 (%) and P).
| Category | Size (6) | | | Localization (8) | | Stage (8) | | Radiotherapy (7) | | Met (11) | | Rec (7) | | CR(5) | | TIL (12) | | CD8+ TIL (7) | |
| --- | --- | --- | --- | --- | --- | --- | --- | --- | --- | --- | --- | --- | --- | --- | --- | --- | --- | --- | --- |
| Bone sarcoma Non- Bone sarcoma | | | | 73.00 35.10 | 0.05 0.17 | 59.60 55.70 | 0.08 0.06 | 70.90 41.60 | 0.00 0.16 | | | 0.00 82.00 | 0.95 0.00 | 68.50 0.00 | 0.04 0.50 | 31.00 75.20 | 0.24 0.00 | 0.00 73.3 | 0.77 0.01 |
| Year ≥ n Year < n | 70.8a1 0.00 | | 0.02 0.63 | 52.70b1 27.70 | 0.08 0.25 | 65.90c1 65.80 | 0.03 0.03 | 0.00d1 81.90 | 0.70 0.00 | 70.80e1 22.40 | 0.00 0.27 | 74.50f1 0.00 | 0.01 0.89 | 96.00g1 47.00 | 0.00 0.15 | 81.40h1 52.00 | 0.00 0.05 | 88.10i1 9.50 | 0.00 0.35 |
| Asian Non- Asian | 70.40 0.00 | | 0.02 0.40 | 64.90 0.00 | 0.01 0.75 | 69.90 0.00 | 0.01 0.83 | | | 84.50 43.00 | 0.00 0.14 | 82.00 0.00 | 0.00 0.95 | 93.20 0.00 | 0.00 0.75 | 62.60 69.20 | 0.03 0.00 | 0.00\* 82.2 | 0.41 0.00 |
| Sample ≥ n Sample < n | 57.20a2 53.7 | | 0.10 0.12 | 53.70b2 59.70 | 0.09 0.06 | 67.80c2 68.60 | 0.03 0.02 | 81.90d2 0.00 | 0.00 0.70 | 80.50e2 63.6 | 0.00 0.02 | 0.00f2 61.5 | 0.80 0.05 | 89.20g2 56.90 | 0.00 0.13 | 81.00h2 45.00 | 0.00 0.11 | 84.90i2 0.00 | 0.00 0.53 |
| Positivity ≥ n (%) Positivity < n (%) | 49.10a3 48.1 | | 0.14 0.15 | 0.00b3 14.60 | 0.38 0.32 | 65.00c3 48.40 | 0.04 0.12 | 0.00d3 46.90 | 0.70 0.15 | 84.90e3 66.20 | 0.00 0.01 | 79.00f3 0.00 | 0.01 0.83 | 89.20g3 56.90 | 0.00 0.13 | 54.30h3 73.80 | 0.05 0.00 | 9.60i3 83.10 | 0.33 0.00 |
| Cut-off ≥ 1% cells Non- ≥ 1% cells | 79.10 0.00 | | 0.01 0.53 | 0.00 14.60 | 0.38 0.32 | 79.30 58.6 | 0.01 0.05 | 46.90 0.00 | 0.15 0.70 | 86.40 61.9 | 0.01 0.01 | 73.00 0.00 | 0.01 0.94 | | | 57.40 78.60 | 0.04 0.00 | 0.00 75.80 | 0.52 0.02 |
| TC + Non-TC + | 57.20 53.70 | | 0.10 0.12 | 60.80 41.10 | 0.11 0.13 | 67.50 35.60 | 0.01 0.21 | 66.00 0.00 | 0.03 0.99 | 64.80 75.70 | 0.02 0.00 | | | 0.00 68.50 | 0.50 0.04 | 78.90 0.00 | 0.00 0.42 | 73.30 0.00 | 0.01 0.77 |
| Total | 54.40 | 0.05 | | 52.30 | 0.04 | 58.50 | 0.02 | 66.60 | 0.01 | 75.80 | 0.00 | 51.30 | 0.06 | 86.70 | 0.00 | 68.90 | 0.00 | 70.10 | 0.00 |
Met: metastasis, Rec: recurrence, CR: chemotherapy response, TIL: tumor-infiltrating lymphocyte, IHC: immunohistochemistry, TC: tumor cell; a1: n = 2017; a2: n = 60; a3: n = 25; b1: n = 2017; b2: n = 90; b3: n = 45; c1: n = 2018; c2: n = 100; c3: n = 60; d1: n = 2018; d2: n = 80; d3: n = 30; e1: n = 2017; e2: n = 90; e3: n = 42; f1: n = 2018; f2: n = 60; f3: n = 22; g1: n = 2018; g2: n = 70; g3: n = 40; h1: n = 2017; h2: n = 60; h3: n = 30; i1: n = 2017; i2: n = 50; i3: n = 20; *: USA vs. Non-USA
